# Mathematical Model of Metabolism and Electrophysiology of Amino Acid and Glucose Stimulated Insulin Secretion: *In Vitro* Validation Using a β-Cell Line

**DOI:** 10.1371/journal.pone.0052611

**Published:** 2013-03-08

**Authors:** Manuela Salvucci, Zoltan Neufeld, Philip Newsholme

**Affiliations:** 1 School of Biomolecular and Biomedical Sciences, University College Dublin, Dublin, Ireland; 2 School of Mathematics and Physics, University of Queensland, Brisbane, Australia; 3 School of Biomedical Sciences, Curtin University, Perth, Australia; Governmental Technical Research Centre of Finland, Finland

## Abstract

We integrated biological experimental data with mathematical modelling to gain insights into the role played by L-alanine in amino acid-stimulated insulin secretion (AASIS) and in D-glucose-stimulated insulin secretion (GSIS), details important to the understanding of complex β-cell metabolic coupling relationships. We present an ordinary differential equations (ODEs) based simplified kinetic model of core metabolic processes leading to ATP production (glycolysis, TCA cycle, L-alanine-specific reactions, respiratory chain, ATPase and proton leak) and Ca^2+^ handling (essential channels and pumps in the plasma membrane) in pancreatic β-cells and relate these to insulin secretion. Experimental work was performed using a clonal rat insulin-secreting cell line (BRIN-BD11) to measure the consumption or production of a range of important biochemical parameters (D-glucose, L-alanine, ATP, insulin secretion) and Ca^2+^ levels. These measurements were then used to validate the theoretical model and fine-tune the parameters. Mathematical modelling was used to predict L-lactate and L-glutamate concentrations following D-glucose and/or L-alanine challenge and Ca^2+^ levels upon stimulation with a non metabolizable L-alanine analogue. Experimental data and mathematical model simulations combined suggest that L-alanine produces a potent insulinotropic effect via both a stimulatory impact on β-cell metabolism and as a direct result of the membrane depolarization due to Ca^2+^ influx triggered by L-alanine/Na^+^ co-transport. Our simulations indicate that both high intracellular ATP and Ca^2+^ concentrations are required in order to develop full insulin secretory responses. The model confirmed that K^+^_ATP_ channel independent mechanisms of stimulation of intracellular Ca^2+^ levels, via generation of mitochondrial coupling messengers, are essential for promotion of the full and sustained insulin secretion response in β-cells.

## Introduction

Pancreatic β-cells have been the subject of both experimental and theoretical interest for several decades as they play a key role in D-glucose homeostasis by adjusting insulin secretion according to blood D-glucose and other nutrients, endocrine and autocrine secretagogues [1], [2]. Insulin release is tightly controlled through complex metabolic and signal transduction relationships in the β-cell. An understanding of the biochemical mechanisms underlying stimulus-secretion coupling in the β-cell is of importance in determining normal and pathogenic (dys)-regulation of insulin secretion in diabetes.

The current hypothesis of the mechanism of D-glucose-stimulated insulin secretion (GSIS) is that D-glucose enters the β-cell via a membrane-bound D-glucose transporter (GLUT1 or GLUT2) where it is metabolized in the pathway of glycolysis, resulting in pyruvate, which can then enter the mitochondria. Pyruvate is oxidized through the Tricarboxylic Acid (TCA) cycle producing reducing equivalents (NADH and FADH_2_) that are transferred to the mitochondrial electron transport chain (ETC), resulting in ATP generation. The rise in the ATP/ADP ratio closes the ATP-sensitive K^+^-channels (K^+^_ATP_) in the cell membrane leading to depolarization, influx of extracellular Ca^2+^ through the voltage-dependent Ca^2+^ (Ca^2+^_ΔΨ_) channels and mobilization of the insulin-containing vesicles causing their fusion with the plasma membrane and release of their cargo [3].

Although the K^+^ ATP–dependent pathway constitutes the main trigger for insulin exocytosis, metabolic coupling factors generated by mitochondrial metabolism, such as nucleotides (ATP, GTP, cAMP, NADPH) and metabolites (malonyl-CoA, citrate, L-glutamate), can markedly affect the full development of insulin secretion [1], [3], [4].

Amino acids represent a significant class of insulin secretion modulators as they are obtained from dietary proteins as well as being released by intestinal epithelial cells [2]. Pancreatic β-cells express a range of specific amino acid transporters, such as systems A, ASC and L – many of which are Na^+^-dependent [5]–[8], allowing amino acids to be rapidly taken up by β-cells. A mixture of physiologic concentrations of amino acids (0.1–0.2 mmol/l) or high concentrations of single amino acids (10–20 mmol/l) have been shown to acutely and chronically modulate insulin secretion both *in vitro* and *in vivo* [7]. It is believed that amino acids may induce insulin secretion by (i) a direct depolarization of the plasma membrane via transport of positively charged amino acids (e.g L-arginine) [7],[9],[10], (ii) metabolism through both triggering and amplifying pathways linked to the TCA cycle (L-glutamine, L-leucine and L-alanine) [3],[5–[8],[10]–[16] and (iii) depolarization of the plasma membrane induced by Na^+^ co-transport (L-alanine and L-proline) [10],[17],[18].

The amino acid L-alanine is one of the most abundant amino acids in plasma (0.5–0.7 mmol/l) [7] in physiological conditions and alterations in its levels, which are indeed found in blood and urine of type two diabetic patients [19]–[21], may significantly affect insulin release.

A number of mathematical and computational models of the GSIS have been developed, although none of them has taken AASIS into account. In particular, modelling efforts have been focussed on the time dependent oscillatory behaviour of the insulin secretion, both of mitochondrial and electrical origin, which are lost in type two diabetes [22]–[24].

Magnus and Keizer built the first combined model of metabolism and Ca^2+^ handling in β-cells focussed on describing the mechanism underlying oscillations rather than the regulatory properties of the network [25]–[27]. The model couples Ca^2+^ handling by the mitochondria with metabolism through ADP and membrane potential. It takes into account transport processes of the inner mitochondrial membrane (redox proton pump, F_1_F_0_-ATPase, proton leak ATP/ADP anti-translocation), the Ca^2+^ uniporter and Na^+^/Ca^2+^ exchanger. A simplified model of GSIS, constituted by four ordinary differential equations for NADH, ADP, membrane potential and Ca^2+^ with pyruvate as input and ATP production as output, has been developed [28] on the basis of the earlier models of Magnus and Keizer. It investigates the effects of Ca^2+^ influx and glycolytic flux on the dynamics of the system and was able to capture the most noticeable features of the Magnus and Keizer model, using a simplified mathematical description. More recently, Fridlyand and co-workers [29] proposed an integrated kinetic model for D-glucose sensing which includes the dynamical description of ADP, NADH, glyceraldehyde 3-phosphate, membrane potential, NADH, Ca^2+^ and pyruvate. The model has been used to investigate the biochemical regulation and control of β-cells, such as alteration of the proton leak activity, NADH shuttles function and reactive oxygen species production.

Since the GSIS machinery is highly complex, mathematical models have also been developed separately for specific sections of the pathway such as metabolism and electrophysiology.

Early stage models of mitochondrial energy metabolism include the work of Garfinkel [30] and Bohnensack [31]. A more detailed model of general mitochondrial metabolism (e.g. non pancreatic β-cell specific) including the respiratory chain, the TCA cycle, fatty acid β-oxidation and the inner membrane transport system, consisting of 58 enzymatic reactions, 117 metabolites and 5 compartments, was assembled using enzymatic kinetic data from different animal sources [32]. This model was integrated with a glycolytic model developed for yeast [33] and constituted the basis for a very complex and detailed kinetic model of metabolic processes in pancreatic β-cell made up of 44 enzymatic reactions, 59 metabolic state variables and 272 parameters [34]. This model could reproduce oscillations in glycolytic metabolites and predict a dose-response curve for ATP for increasing concentrations of D-glucose. Other pancreatic β-cell-specific models have been developed for specific parts of the pathway, such as NADH shuttles [35] and the initial section of glycolysis [35],[36]. This glycolytic model underlines the dependency of the occurrence of oscillations as a function of glucokinase, aldolase, phosphofructokinase and GAPD activities. Other detailed models for TCA cycle [37] and oxidative phosphorylation [38] in specific tissues such as heart, muscle and liver have previously been developed. In these models the number of equations and parameters necessary to describe the dynamic of the system is very high and this may obscure coupling mechanisms and make the mathematical interpretation difficult.

Most current models of electrical activity in pancreatic β-cells focus on explaining the complex bursting behaviour. The bursting consists of an active phase when depolarization of the plasma membrane causes Ca^2+^ to flow into the cell, alternating with a silent phase when Ca^2+^ is extruded and the membrane potential repolarizes. The cyclic alternation between depolarization and repolarization of the plasma membrane leads to oscillations in cytosolic Ca^2+^ concentrations [29],[39]–[44].

The mechanism of the bursting of electrical activity and Ca^2+^ oscillations is based on the separation of timescales: a fast sub-system, constituted by the membrane potential and delayed rectifier potassium channels, generate the spiking during the active phase, whereas a slow variable provide the negative feedback responsible for the cyclical depolarization/repolarization [29],[39]–[44].

**Figure 1:**
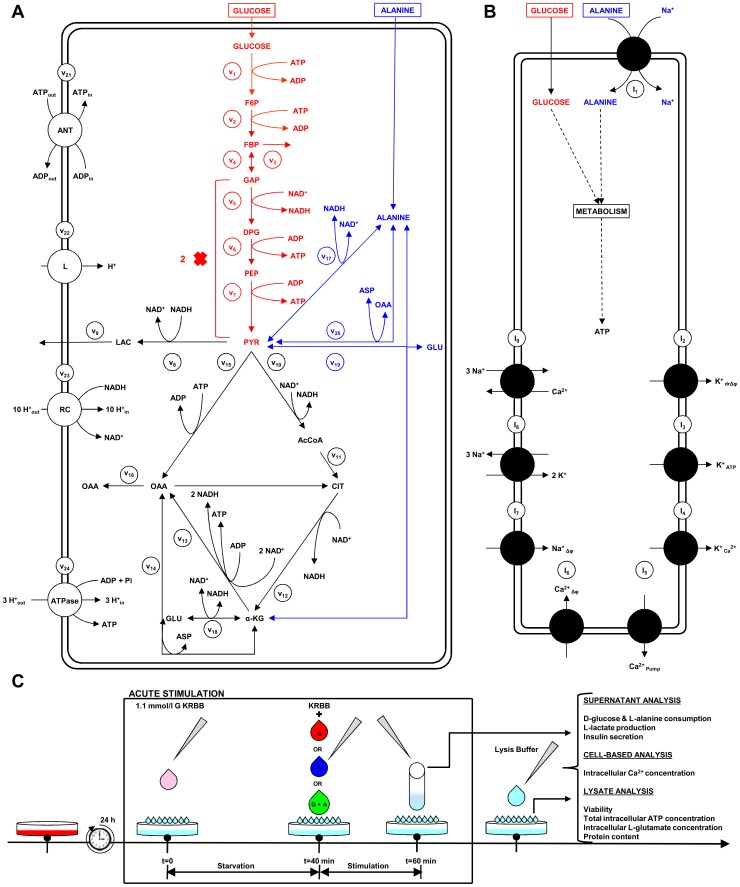
GSIS and AASIS machinery in the pancreatic β-cell. **A. Schematic diagram of metabolic processes accounted for in model 1.** Reactions are represented as arrows (either uni- or bidirectional) and labelled 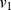
to 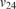 (**Table 2**). Red arrows represent D-glucose specific pathways, blue arrows indicate L-alanine-related reactions whereas black arrows denote viable metabolic routes common to both D-glucose and L-alanine. **B. Schematic representation of downstream electrophysiologic events and ion fluxes included in model 2**. In clockwise order: Na^+^/L-Alanine Co-transport (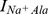), delayed rectifying K+ current (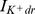), K+ ATP-dependent current (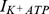), K+ Ca^2+^-activated current (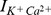), Ca^2+^ plasma membrane pump (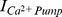), Ca^2+^ Uniporter (voltage-dependent) current (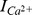), Na+ voltage-gated current (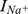), Na+/K+ pump current (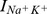), Na+/Ca^2+^ exchanger current (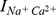). Current equations are given in **Table 3** **. C. Experimental workflow.** BRIN-BD11 cells were washed with pre-warmed PBS and starved at 37°C for 40 min in 1.1 mmol/l D-glucose KRBB. The cells were then washed again with PBS and stimulated for 20 minutes in KRBB supplemented with different concentrations of D-glucose (G) only (1.1, 5, 16.7 and 30 mM), L-alanine (A) only (0.5, 1, 2, 5 and 10 mM) or their combination (G + A). After incubation, an aliquot of supernatant was removed for later quantification of D-glucose and L-alanine consumption, L-lactate production and insulin secretion. Intracellular 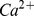
concentration was assessed by flow cytometry. Cells were then washed with ice-cold PBS and lysed to assess viability, intracellular ATP concentration, intracellular L-glutamate concentration and protein content. (*) Different lysis buffers were used depending on the biochemical parameter being measured.

The main goal of this work is to provide new insights on the role played by L-alanine in amino acid stimulated insulin secretion (AASIS) and the potent enhancing effect on D-glucose-stimulated insulin secretion (GSIS), as a first step to understanding the complex metabolic relationships in the β-cell. We consider the insulin secretion pathway divided in two main blocks: (i) core metabolic network leading to ATP production (model 1) (**Figure 1. A**) and (ii) electrical activity of ATP concentration-driven channels that leads to Ca^2+^ influx resulting in insulin granule exocytosis (model 2) (**Figure 1. B**), as detailed in the Materials and Methods section.

Our model of GSIS and AASIS in the β-cell was validated against wet-lab observations carried out on a functional clonal rat insulin secreting β-cell line (BRIN-BD11) [45]. An *ad hoc* experimental procedure was designed to obtain both single (D-glucose or L-alanine) and combined (D-glucose + L-alanine) acute stimulus dose-response curves (**Figure 1. C**).

We are interested in modelling the mechanisms of possible metabolic stimulus-coupling effects rather than the time-dependent behaviour of the system. Thus, all simulations were allowed to run until the steady state was reached and all presented experimental results were recorded after 20****minutes incubation with stimuli of interest.

## Results

### Effect of D-glucose and L-alanine on cell integrity and insulin secretory responses

BRIN-BD11cells integrity was investigated by neutral red assay following 1****h incubation in Krebs Ringer Bicarbonate Buffer (KRBB) supplemented with either various concentrations of D-glucose (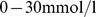) in the presence or absence of 10 mmol/l L-alanine or different concentrations of L-alanine (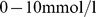) with/without supplementation with 16.7 mmol/l D-glucose. These experiments were performed to ensure that cells viability was not compromised under the incubation conditions tested (**Table 1**).

**Table 1:** BRIN-BD11 cells viability following 1 h incubation in stimuli-supplemented KRBB.

	10 mmol/l L-alanine	
D-glucose concentration [mmol/l]	−	+
**0.0**	87.1±8.9 *	110.2±3.4
**1.1**	97.2±3.4	113.2±3.6
**5.0**	99.1±5.2	107.7±3.2
**11.1**	100.0±4.3	105.3±4.5
**16.7**	105.6±4.5	106.2±5.2
**30.0**	103.3±5.2	102.1±3.5

	16.7 mmol/l D-glucose	
L-alanine concentration [mmol/l]	−	+
**0.0**	87.1±8.9^+^	105.6±4.5
**0.5**	98.0±3.1	103.0±4.7
**1.0**	102.0±2.3	99.4±3.5
**2.0**	101.8±2.8	97.9±5.6
**5.0**	107.0±5.6	101.0±3.2
**10.0**	110.2±3.4	106.2±5.2

BRIN-BD11 cells were incubated for 1 h in KRBB supplemented with either various concentrations of D-glucose (0–30 mmol/l) in the absence or presence of 10 mmol/l L-alanine or L-alanine (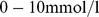) with/without supplementation with 16.7 mmol/l D-glucose. Results are expressed as percentage of control (11.1 mmol/l D-glucose, same concentration of RPMI-1640 media used to maintain cells in culture). Data are mean ± SD (n = 3).^ *^ 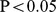 stimuli-free KRBB *vs* 16.7 mmol/l D-glucose KRBB, ^+^ 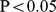 stimuli-free KRBB *vs* 10 mmol/l L-alanine.

**Figure 2:**
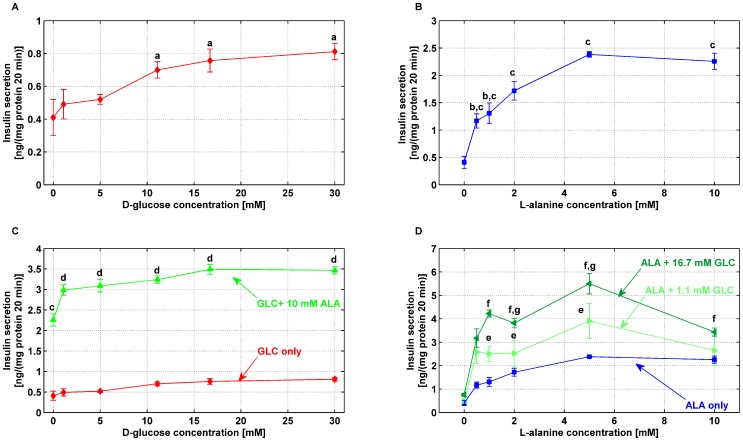
Effects of D-glucose and L-alanine on acute insulin secretion from BRIN-BD11 cells. BRIN-BD11 cells were cultured, allowed to adhere overnight prior to being pre-incubated (40 min) in 1.1 mmol/l D-glucose and the acute (20 min) insulinotropic effects of D-glucose (GLC) only (**A**), L-alanine (ALA) only (**B**) and combinations of both substrates (**C, D**) were tested. Values are mean ± SD of at least 3 independent experiments. In **A**, ^a^ 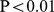 *vs* basal (1.1 mmol/l) D-glucose KRBB; in **B**, ^b^ 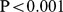 *vs* stimuli-free KRBB,^ c^ 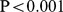 *vs* basal (0.5 mmol/l) L-alanine KRBB; in **C**, ^c^ as in B, ^d^ 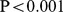 *vs* absence of 10 mmol/l L-alanine; in (**D**), ^e^ 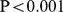 *vs* absence of 1.1 mmol/l,^ f^ 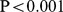 *vs* absence of 16.7 mmol/l D-glucose and ^g^ 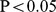 L-alanine KRBB supplemented with 1.1 mmol/l D-glucose compared to same concentrations supplemented with 16.7 mmol/l D-glucose.

Acute insulin secretion was concentration dependent with respect to D-glucose only, rising over the range 1.1 mmol/l to 30 mmol/l from 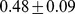 ng/(mg protein 20 min) to 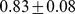 ng/(mg protein 20 min) (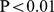), (**Figure 2. A**). Administration of L-alanine only exhibited a robust dose-dependent insulin secretion, increasing from 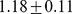 ng/(mg protein 20 min) to 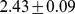 ng/(mg protein 20 min) (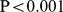) over the range 0.5 mmol/l to 10 mmol/l, (**Figure 2. B**). L-alanine and D-glucose combined insulinotropic effects are non trivial: addition of 10 mmol/l L-alanine to 1.1–30 mmol/l D-glucose did not result in just an additive insulin secretion, but rather in a synergistic increase (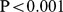) in GSIS (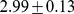 ng/(mg protein 20 min) and 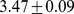 ng/(mg protein 20 min) at 1.1 and 30 mmol/l, respectively), (**Figure 2. C**). Similarly addition of D-glucose both at basal (1.1 mmol/l) and stimulatory (16.7 mmol/l) concentrations to 0.5–10 mmol/l L-alanine led to a significant increase (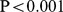) in insulin secretion (**Figure 2. D**).

### Effect of D-glucose and L-alanine on ATP and L-lactate concentrations

An increase in ATP concentration is the main triggering event in the K^+^ ATP-dependent pathway of nutrient-elicited insulin secretion. Thus, our first goal was to build a simplified mathematical model for ATP production (model 1), which could reproduce experimental ATP data following D-glucose and/or L-alanine stimulation.

In order to estimate the input fluxes for model 1 corresponding to the range of D-glucose and L-alanine concentrations administered in the experiments (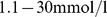 D-glucose and 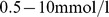 L-alanine), stimuli consumption assays were performed.

**Figure 3:**
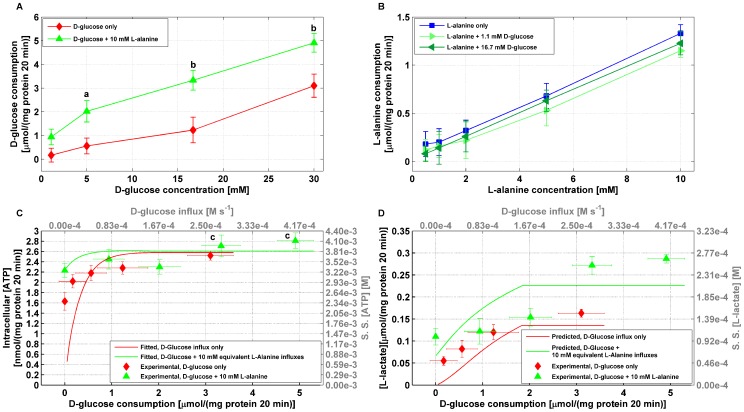
Effects of D-glucose and L-alanine on ATP and L-lactate production. All experiments were performed following 40 min pre-incubation in 1.1 mmol/l D-glucose and 20 min acute stimulation. Values are mean ± SD of at least 3 independent experiments. **A.** Experimental D-glucose consumption as a function of D-glucose concentration. ^a^ 5 mmol/l D-glucose *vs* 5 mmol/l D-glucose plus 10 mmol/l L-alanine (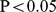), ^b^ D-glucose *vs* same concentration of D-glucose supplemented with 10 mmol/l L-alanine (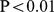). **B.** Experimental L-alanine consumption as a function of L-alanine concentration. **C**. Experimental total intracellular ATP concentration as a function of D-glucose influx in the absence or presence 10 mmol/l L-alanine (red diamonds and green triangles, respectively). ^c^ D-glucose *vs* addition of 10 mmol/l L-alanine (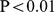). Simulated steady state ATP concentrations as a function of D-glucose influx (red and green solid lines) were fitted with a least square criterion to ATP observations by adjusting the parameters 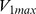, 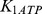, 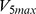, 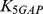, 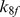, 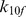, 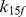, 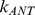, 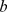 and 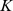. **D.** Experimental L-lactate concentrations as a function of D-glucose influx in the absence or presence of 10 mmol/l L-alanine (red diamonds and green triangle, respectively). ^d^ 16.7 mmol/l *vs* 30 mmol/l D-glucose (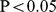), ^e^ 5 mmol/l D-glucose *vs* 5 mmol/l D-glucose plus 10 mmol/l L-alanine (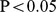) and ^f^ D-glucose *vs* addition of 10 mmol/l L-alanine (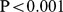). Predicted steady state L-lactate concentrations (red and green solid lines) were simulated using the standard set of parameters listed in Table S1 that were obtained by fitting to ATP observations in **panel C**.

D-glucose consumption increased dose-dependently with respect to D-glucose in the range (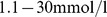) from 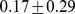 µmol/(mg protein 20 min) to 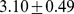 µmol/(mg protein 20 min), (**Figure** **3. A**). Analogously, L-alanine consumption exhibited a dose-response increase from 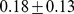 µmol/(mg protein 20 min) to 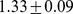 µmol/(mg protein20 min) in the range 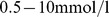 L-alanine (**Figure 3. B**). Addition of 10 mmol/l L-alanine to D-glucose enhanced D-glucose consumption (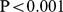) whereas L-alanine consumption was found unaffected by the presence of D-glucose at both basal (1.1 mmol/l) and stimulatory (16.7 mmol/l) concentrations (**Figure 3. A–B**).

Experimental ATP concentration changes obtained over a 20 min incubation period were found dose-dependent with respect to D-glucose: increasing D-glucose in the range 1.1 mmol/l to 30 mmol/l increased ATP content from 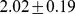 nmol/(mg protein 20 min) to 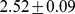 nmol/(mg protein 20 min). Addition of 10 mmol/l alanine slightly increased ATP production compared to D-glucose only, which became significant (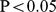) at 16.7 mmol/l (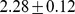 nmol/(mg protein 20 min) *vs* 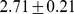 nmol/(mg protein 20 min)) and 30 mmol/l (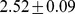 nmol/(mg protein 20 min) *vs* 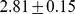 nmol/(mg protein 20 min)). 10 mmol/l. L-alanine administered on its own led to an ATP production of 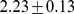 nmol/(mg protein 20 min), comparable to the value achieved with stimulatory concentrations of D-glucose (**Figure 3. C**).

In order to fit the computational steady state 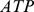 concentrations to the experimental observations, a least-squared optimization criterion was used to adjust a subset of the kinetic parameters (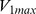, 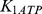, 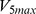, 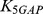, 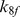, 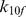, 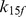, 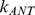, 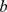 and 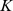). The optimization procedure was set to minimize the objective function (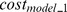) given by the sum over the 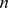 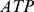 observations of the squared normalized difference upon experimental 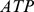 data (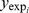) and simulated steady state 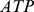 concentrations (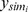), (1).
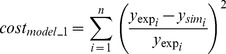
(1)


The optimization was implemented in MATLAB with the routine *fmincon* with lower and upper bounds set to 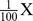 and 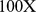 of the initial values. As initial values, we used the parameters reported in [33],[46]. Note that the fitting has been carried out on only 9 of the 10 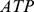 observations since the model is intended to simulate 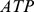 production following input with D-glucose or L-alanine and not the basal 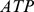 production/consumption of the cell.

The results of the fitting are shown in **Figure 3. C** (solid red and green lines for D-glucose only without/with applying 10 mmol/l equivalent L-alanine flux, respectively) and are in good agreement with the experimental data (red diamonds and green triangles for D-glucose in the absence/presence of 10 mmol/l L-alanine, respectively).

In order to obtain a good fit, it was critical to adjust the parameter 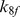 (reaction 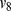 in **Figure 1. A**), which represents the forward kinetic constant for conversion of pyruvate into L-lactate via lactate dehydrogenase. Pancreatic β-cells are characterized by very low activity of lactate dehydrogenase, however clonal β-cell lines exhibit higher L-lactate outputs with respect to primary cells [47],[48]. Indeed, we found that a high value of 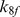 was needed to prevent a decrease in the steady state 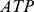 concentrations towards higher D-glucose input fluxes, especially if applied in conjunction with L-alanine. Following a step increase in D-glucose input, the overall flux along the glycolytic pathway leading to pyruvate increases. If 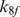 is set to zero, the relative flux (relative to the sum of all fluxes responsible for pyruvate breakdown) through reaction 15 (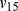) increases and the relative flux through reaction 10 (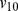) decreases as a function of D-glucose influx.

**Figure 4:**
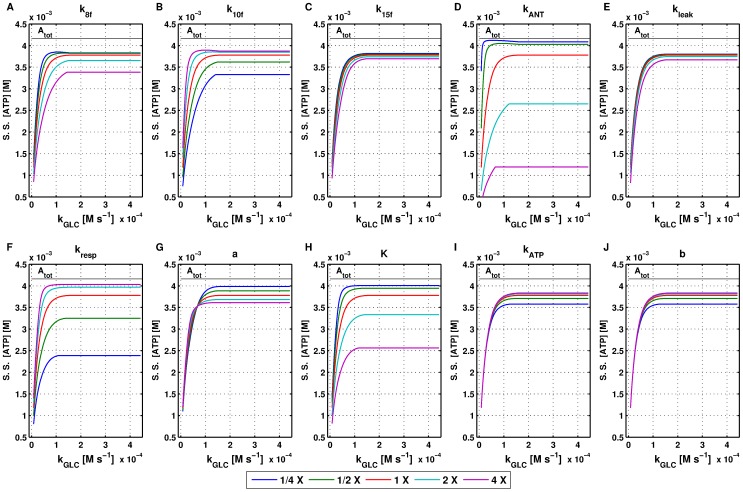
Steady state 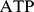 concentration sensitivity analysis for mathematical model 1. All simulations were performed using mathematical model 1 with standard parameters listed in **Table S1**. Parameters were varied individually by a factor of 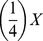, 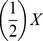, 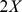, 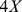 and the resulting steady state 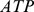 concentrations as a function of D-glucose influx are shown.

A counterintuitive result of the simulations is that shifting to L-lactate production by increasing 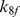 has the twofold advantage of leading to 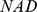 production which can serve as a further supply for the glycolysis and redirecting some pyruvate that otherwise would have been metabolised through reaction 15 (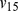) consuming 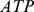 (see also **Figure 4. A–C**). Overall, this leads to progressive lower steady state 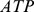 concentrations across the whole D-glucose input flux range. However, this results in a monotonic increasing behaviour as the one observed experimentally (**Figure 3. C**).

For 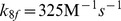, as found by fitting computational 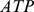 to experimental 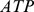, the model predicts a marked L-lactate output when D-glucose influx is applied and an even more robust levels when L-alanine input is also present (solid red and green lines, respectively in Figure 3. D). These results were confirmed by experimental observations (red diamonds and green triangles, respectively in Figure 3. D). L-lactate production increased dose-dependently with respect to D-glucose from 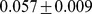 µmol/(mg protein 20 min) to 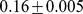 µmol/(mg protein 20 min) in the range (1.1 to 30 mmol/l D-glucose) and was further enhanced by supplementation with 10 mmol/l L-alanine (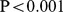). Marked L-lactate output in BRIN-BD11 cells following acute stimulation with D-glucose has also been reported in other studies [9],[15],[49].

Model 1 is characterized by 41 adjustable parameters and 10 of them (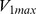, 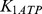, 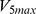, 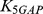, 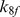, 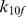, 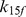, 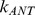, 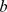, 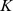) were used to fit the computational 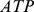 data to the 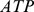 experimental observations. This subset of parameters was selected based on sensitivity studies. Sensitivity is defined as the relative change of the output variable (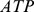 in this case) due to a relative change of one parameter. We were interested in identifying which parameters could singularly shape the steady state 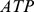 concentration. Thus, we varied the value of one parameter at the time by factors 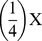, 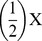 and 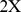, 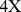, keeping the rest of the parameters at their default values. We then visually inspected the resulting curves looking for either increase/decrease in concentration or variation in behaviour across increasing input flux.

Steady state ATP concentration is also sensitive to the values assumed by some parameters related to the glycolytic pathway (namely 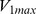, 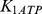, 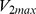, 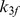, 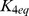, 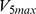, 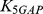 and 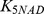).

**Figure 4** shows the sensitivity analysis results for mitochondria-related parameters as a function of D-glucose influx. It is well established that the distribution of TCA fluxes determines the status of the oxidative state. This is coupled with the respiratory activity, which in turn affects both ATP synthase and ATP/ADP translocation. Therefore, it is not surprising that simulations indicated that steady state 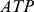 concentration is sensitive to the values of some specific parameters linked to either the distribution of fluxes in the TCA cycle or the respiratory chain and ATP synthase rates. For example, **Figure 4. A–C** show how the kinetic constants that catalyse pyruvate breakdown, either by entering TCA cycle through both reaction 10 and reaction 15 or disposal through L-lactate (reaction 8), have a critical importance on steady state 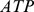 concentration. Increasing 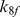 results in both a slower rise and a lower saturation level for 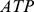 concentration as a function of D-glucose influx (**Figure 4. A**). Conversely, increasing the kinetic constant 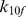
leads to 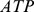 increasing more steeply as a function of D-glucose influx and culminating in higher steady state level (**Figure 4. B**). The kinetic constant 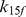 also slightly contributes to lowering the steady state 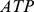 concentrations if increased.

The kinetic constant for ATP translocation has a significant impact on steady state 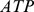 concentrations. 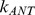 represents the activity of ATP/ADP carrier and accounts for the energetic status of the cell (state 3, 3.5 and 4, [46]). Higher 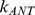 values result in a marked reduction in steady state 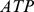 concentrations due to a decrease in both respiration and ATP synthase rates (**Figure 4. D**).

The kinetic constant for proton leak across the mitochondrial membrane (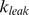) can also affect steady state 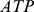 concentration especially when increased (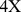, purple solid line), (**Figure 4. E**).

Other parameters that are critical in 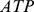 concentration are involved in the respiratory activity (**Figure 4. F–H**). The respiratory rate is described by a differentiable function, which has a saturation curve for 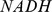 and behaves almost linearly with respect to 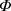 until it reaches the threshold 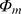, after which it rapidly approaches zero. Varying the parameter 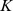
affects the saturation curve for 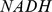: the quantity 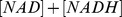 is conserved and equal to 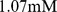 whereas 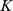
is equal to 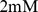 in the original study [46] and was adjusted to 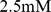 in the 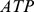 fitting. Thus, for the limit case where all 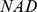 is converted into 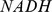 (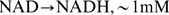) selecting a 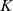 in the same range will produce a linear increase in the respiratory rate with respect to 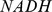 (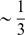
if 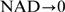 and 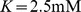) which will tend to zero as 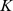
increases, resulting in lower 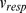 and consequently in a reduction in 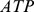 concentration.

Another viable way to boost the steady state 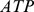 concentrations is by increasing the parameters involved in the rate for ATP synthase (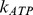 and 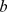 in 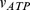, reaction 24), (**Figure 4. I–J**).

### Effect of D-glucose and L-alanine on L-glutamate concentration

**Figure 5:**
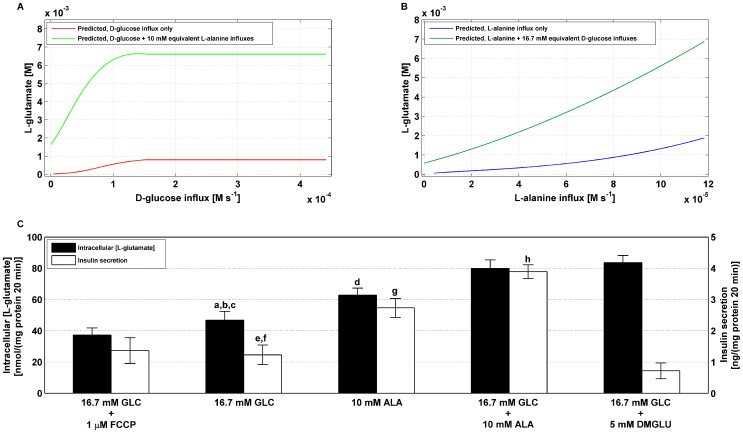
Relationship between intracellular L-glutamate levels and insulin output. **A.** Simulated steady state intracellular L-glutamate concentrations produced in response to a step increase in D-glucose influx in the absence (solid red line) and presence of a fixed L-alanine input equivalent to 10 mmol/l concentration administered in experiments (solid green line). **B.** Simulated intracellular L-glutamate concentrations as a function of L-alanine input flux only (solid blue line) and with a fixed D-glucose input equivalent to 16.7 mmol/l concentration administered in the experiments (solid dark green line). Both in **A** and **B** simulations results were obtained using mathematical model 1 with parameter values listed in **Table S1**. **C.** BRIN-BD11 cells were cultured, allowed to adhere over a 24 h period prior to being pre-incubated (40 min) in 1.1 mmol/l D-glucose, acutely stimulated for 20 min with either D-glucose only (16.7 mmol/l), L-alanine only (10 mmol/l), combinations of both substrates, 16.7 mmol/l D-glucose supplemented with 5 mmol/l DMGLU and 16.7 mmol/l D-glucose plus 1 µmol/l FCCP. Supernatant was assayed for insulin secretion and lysates were analysed for intracellular L-glutamate content. Values are mean ± SD of at least 3 independent experiments. Statistical significance: **L-glutamate:** ^a^ 16.7 mmol/l D-glucose *vs* 16.7 mmol/l D-glucose plus FCCP (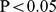), ^b^ 16.7 mmol/l D-glucose *vs* 16.7 mmol/l D-glucose supplemented with 10 mmol/l L-alanine (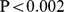),^ c^ 16.7 mmol/l D-glucose *vs* 16.7 mmol/l D-glucose plus 5 mmol/l DMGLU (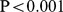), ^d^ 10 mmol/l L-alanine *vs* 16.7 mmol/l D-glucose plus 10 mmol/l L-alanine (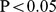).**Insulin:** ^e^ 16.7 mmol/l D-glucose *vs* presence of 10 mmol/l L-alanine (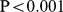), ^f^ 16.7 mmol/l D-glucose *vs* addition of DMGLU (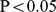), ^g^ 10 mmol/l L-alanine *vs* supplementation with 16.7 mmol/l D-glucose (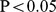), ^h^ 16.7 mmol/l D-glucose plus 10 mmol/l L-alanine *vs* 16.7 mmol/l D-glucose supplemented with 5 mmol/l DMGLU (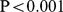).

Model 1 simulations predicted an increase in the steady state intracellular L-glutamate concentrations for increasing D-glucose influx only and a marked shift upward of the curve when L-alanine input was applied in combination (**Figure 5. A–B**). This is mainly due to an increase in the fluxes through reactions 18 (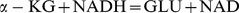) and 19 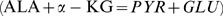, which both almost evenly contribute towards production of L-glutamate. Due to the topology of the network the flux 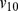 (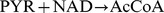) has to be equal in magnitude and sign (direction) to flux 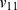 (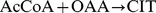) and consequently 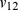 (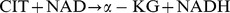). Moreover, flux 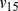 (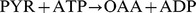) has to balance out flux 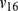 (discard of 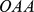). For these reasons, the balance at “node” 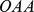 leaves us with flux 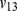 that needs to be equal in magnitude and opposite in sign to flux 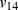, which consumes 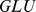 and 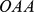 to produce 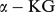 and 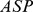. Flux 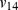 must be balanced by flux through reactions 18 and 19, as previously stated.

Mitochondrial metabolism is known to play a crucial role in nutrient-stimulated insulin secretion not only for the production of ATP, but also for the concomitant generation of stimulus-secretion coupling factors, which are believed to be responsible for the full and sustained insulin secretion. Indeed, L-glutamate is one of the most controversial putative mitochondrial metabolism-derived messengers.

In order to test whether L-alanine exploits its insulinotropic effect through generation of the potential coupling agent L-glutamate, an experiment was specifically designed. The acute effects (20 min) in the presence of a stimulatory concentration of D-glucose (16.7 mmol/l), L-alanine (10 mmol/l), their combination, 16.7 mmol/l D-glucose supplemented with a cell-permeable L-glutamate precursor (5 mmol/l of dimethyl-glutamate, DMGLU) and a negative control (16.7 mmol/l D-glucose plus the mitochondrial poison FCCP, carbonyl cyanide p-trifluoromethoxyphenylhydrazone) on intracellular L-glutamate content and corresponding insulin secretion levels were investigated. DMGLU was successfully utilized in inducing a dose-dependent increase in intracellular L-glutamate (results not shown) and the concentration of 5 mmol/l of DMGLU administered in combination with 16.7 mmol/l D-glucose were found to produce an “artificial” increase in intracellular L-glutamate of 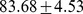 nmol/(mg protein 20 min) comparable in content to 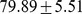 nmol/(mg protein 20 min) induced by 16.7 mmol/l D-glucose plus 10 mmol/l L-alanine. Insulin secretion stimulated by 16.7 mmol/l plus 5 mmol/l DMGLU was slightly, but significantly (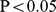), decreased when compared to D-glucose only (**Figure 5. C**) suggesting that the potent stimulus 16.7 mmol/l D-glucose plus 10 mmol/l L-alanine does not exploit its insulinotropic effect primarily through generation of L-glutamate. Addition of 1 µmol/l of the uncoupling poison FCCP successfully inhibited the production of L-glutamate during D-glucose stimulation (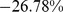 of intracellular L-glutamate, 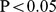), although it did not significantly affect insulin release, further corroborating the hypothesis that L-glutamate content is not directly correlated with insulin secretion.

### Effect of D-glucose and L-alanine on 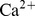 concentration

As shown in **Figure 3. C**, stimulatory concentrations of D-glucose (16.7 mmol/l) and 10 mmol/l of L-alanine resulted in comparable levels of ATP (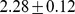 *vs* 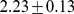 nmol/(mg protein 20 min)), despite their insulin secretion levels differed markedly (**Figure 2. A–B**). These data suggest that some other mechanism beside ATP must be involved in insulin secretion.

L-alanine is taken up by β-cells at very high rate in BRIN-BD11 cells (one order of magnitude higher that of L-glutamine, [16]) and it is co-transported with Na^+^ with a stoichiometric ratio 1∶1. This could lead to a substantial shift in the intracellular Na^+^ concentration, which could affect Ca^2+^ handling and ultimately account for the disparity in insulin secretion.

**Figure 6:**
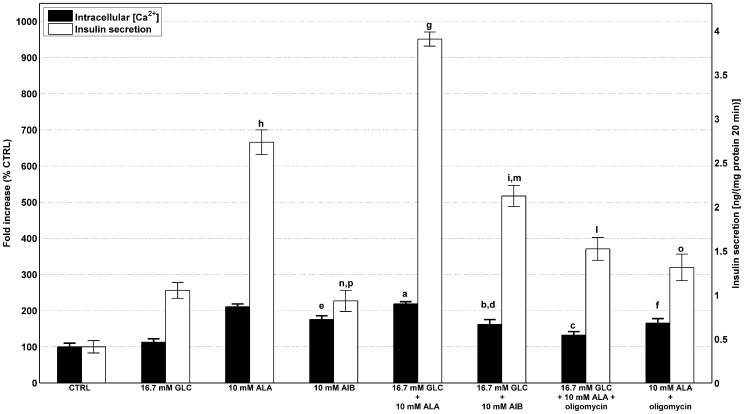
Effects of D-glucose and/or L-alanine on intracellular Ca^2+^ and insulin secretion. BRIN-BD11 cells were cultured, allowed to adhere over a 24 h period prior to being pre-incubated (40 min) in 1.1 mmol/l D-glucose, acutely stimulated for 20 min with either D-glucose only (16.7 mmol/l), L-alanine only (10 mmol/l, with/without 1.8 µg/ml oligomycin), combinations of both substrates with/without oligomycin, 16.7 mmol/l D-glucose supplemented with 10 mmol/l AIB and 10 mmol/l AIB only. Samples were assayed for insulin secretion and intracellular Ca^2+^ concentration as described in the Materials and Methods section. Values are mean ± SD of at least 3 independent experiments. Statistical significance: **Intracellular Ca^2+^ concentration**: ^a^ D-glucose only *vs* addition of 10 mmol/l L-alanine (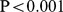), ^b^ D-glucose only *vs* addition of 10 mmol/l AIB (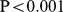), ^c^ D-glucose plus L-alanine *vs* addition of oligomycin (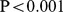), ^d^ D-glucose plus L-alanine *vs* D-glucose plus AIB (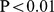), ^e^ L-alanine *vs* AIB (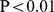), ^f^ L-alanine *vs* addition of oligomycin (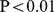). **Insulin secretion**: ^g^ D-glucose *vs* addition of 10 mmol/l L-alanine (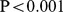), ^h^ D-glucose plus L-alanine *vs* L-alanine only (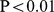), ^i^ D-glucose *vs* addition of 10 mmol/l AIB (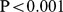), ^l^ D-glucose plus L-alanine *vs* addition of oligomycin (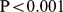), ^m^ D-glucose plus L-alanine *vs* D-glucose plus AIB (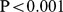), ^n^ L-alanine *vs* AIB (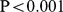), ^o^ L-alanine *vs* addition of oligomycin (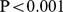), ^p^ D-glucose *vs* addition of AIB (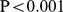).

Intracellular Ca^2+^ concentration responses to acute stimulation with D-glucose (16.7 mmol/l), L-alanine (10 mmol/l) and their combination are presented in **Figure 6**. Intracellular Ca^2+^ concentration responses were found to positively correlate with insulin secretion.

The potent insulinotropic stimulus 16.7 mmol/l D-glucose plus 10 mmol/l L-alanine was associated with a relative fold increase of 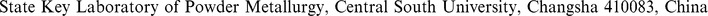 and 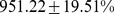 in intracellular Ca^2+^ concentration and insulin secretion respectively against 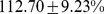 and 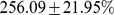 exhibited by D-glucose only and 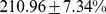 and 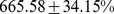 by L-alanine only.

To determine the relative importance of oxidative metabolism and Na^+^ co-transport in L-alanine-induced stimulation of insulin secretion, an experiment was designed whereby either 10 mmol/l of a non-metabolizable L-alanine analogue, AIB (α-aminoisobutyric acid), which shares the same co-transport mechanism of the amino acid, or the mitochondrial poison oligomycin (1.8 µg/ml) were employed.

When 10 mmol/l L-alanine were replaced by equimolar amounts of AIB, either when administered alone or supplemented with 16.7 mmol/l D-glucose, both intracellular Ca^2+^ concentration (from 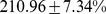 to 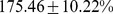, 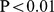 and 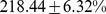 to 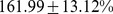, 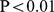, respectively) and insulin release (from 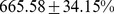 to 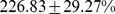, 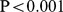 and 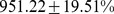 to 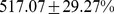, 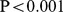, respectively) were markedly decreased. The addition of oligomycin to 10 mmol/l L-alanine with/without D-glucose provoked substantial decrease in both Ca^2+^ levels (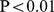) and insulin release (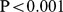). Experimental results substantiated our hypothesis that Na^+^ co-transport may have a significant impact on L-alanine induced insulin secretion. However, Na^+^ co-transport is not the only factor responsible for increased insulin secretion as suggested by the results obtained with 10 mmol/l L-alanine *vs* 16.7 mmol/l plus 10 mmol/l L-alanine. Ca^2+^ level was slightly (from 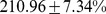 *vs* 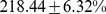), but not significantly, enhanced, while the corresponding insulin secretion was markedly increased (from 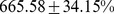 to 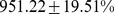).

In order to characterise further the role of amino acid/Na^+^ co-transport on Ca^2+^ dynamics, we built a simplified mathematical model of Ca^2+^ handling, based on the framework of Fridlyand, as described in the Materials and Methods section. The model main input is metabolism-derived ATP production (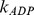, (72)) and it does not distinguish whether the ATP is obtained from D-glucose or L-alanine oxidation.

We then introduced an additional Na^+^ current (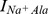,) to model the Na^+^/L-alanine co-transport across the plasma membrane as detailed in the Materials and Methods.

Pancreatic β-cells are characterized by an oscillatory electrical and 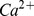 activity upon stimulation resulting in an oscillatory insulin secretion. The electrical activity shows complex patterns and a large body of theoretical and experimental studies have focussed on trying to clarify the mechanism involved [14],[25],[27],[41],[42],[50]–[52].

**Figure 7:**
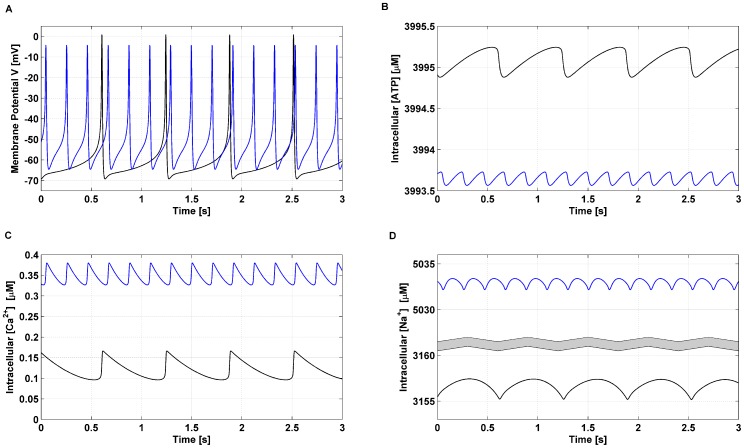
Electrical activity and intracellular concentrations of 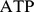 and cations. Time-dependent changes in the membrane potential 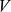 (**A**), intracellular 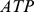 concentration (**B**), intracellular 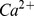 concentration (**C**) and intracellular 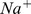 concentration (**D**) are depicted. The simulation with the default parameters given in **Table S2** and 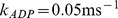 is represented by the solid black curve. The effect of applying 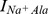 to model Na^+^/L-alanine co-transport was simulated by increasing 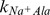 from 0 to 50 in equation 69 (solid blue line).

The time course behaviour for the state variables 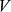, 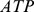, 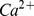 and 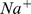 with or without applying 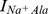 is shown in **Figure 7** (solid blue line and solid black line, respectively). In response to 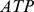 (simulated with standard parameter values listed in **Table S2** and 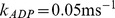), the membrane potential exhibited peaks at 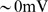 and troughs at 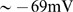 *vs* 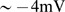 and 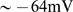 when 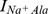 (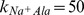) was applied. 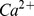, 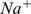 and 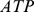 show the typical oscillating pattern, however the frequency was also altered when 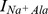 was applied from 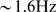 (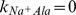) to 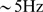 (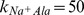).

Metabolism-derived ATP raise was simulated by increasing the rate constant for 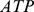 production over a broad range of values (from 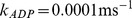 to 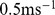, corresponding to basal 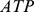 production and virtually no stimuli to a saturating region), while the impact of 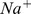 co-transport for increasing concentration of L-alanine was modelled by the adjustable parameter 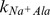 in the range 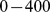. Simulations were run until an oscillatory stationary state was reached and the mean of the state variables and the channels’ currents was computed over time.

**Figure 8:**
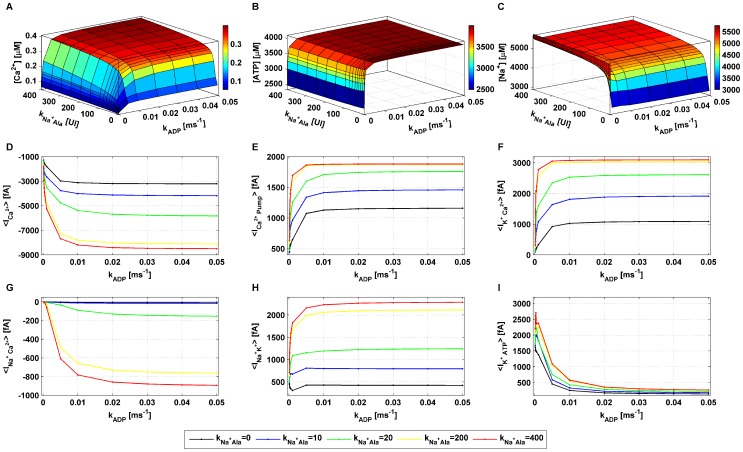
Mean 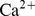, 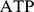, 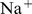 concentrations and main channels’ currents modulated by 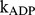 and 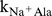. 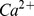
 handling model simulations were run until an oscillatory steady state was reached and subsequently the mean was computed over time for each state variable and current in the system. Standard parameter values listed in **Table S2** were used. The effect of a step increase in 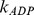 (in the range 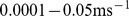) for various values of the parameter 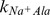 on intracellular 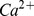, 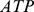, 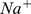 concentrations is reported in **panels A–C.** The effect of a step increase in 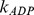 (in the range 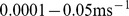) for the parameter 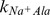 assuming the values 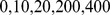 on the main currents included in the model, 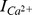, 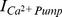, 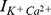, 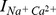, 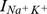, 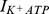 is shown in **panels D–I**.

The effect of a step increase in 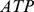 production and 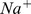 co-transport on steady state intracellular concentrations of 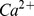, 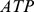 and 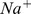 and the main channels currents included in the model, Ca2+ uniporter (voltage-dependent) current (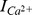), Ca2+ plasma membrane pump (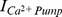), K+ Ca2+-activated current (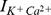), Na+/Ca2+ exchanger current (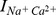), Na+/K+ pump current (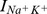), and K+ ATP-dependent current (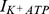), is presented in **Figure 8** **.** A step increase in 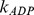 resulted in an increase in the intracellular 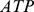 concentration, which caused a decrease in the current flowing through the K^+^ATP-dependent channel. The consequent plasma membrane depolarization led to Ca^2+^ influx through the Ca^2+^ voltage dependent channel as expected.

Applying 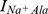 resulted in a substantial increase in the mean 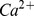 concentration, supporting the hypothesis that the raise in intracellular 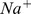 concentration due to the symport may play a significant role in insulin secretion. The intracellular 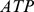 concentration was slightly reduced as the system increases the activity of the 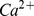 pump and the Na^+^/K^+^ pump to extrude the excess of 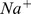 (**Figure 8. E** and **Figure 8. H**, respectively), both ATP-fuelled. Moreover, the raise in 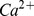 concentration directly accelerates 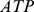 consumption (72). This decrease in 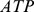 concentration led to a slight increase in the outward current through K^+^ ATP-dependent channel, especially for low 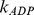 values. However, applying 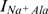 led to a marked increase in the K^+^-Ca^2+^ activated current (**Figure 8. F**) and in the current flowing through the K^+^ delayed rectifier channel (not shown). Along with the Ca^2+^ pump current, the Na^+^/Ca^2+^ exchanger current (**Figure 8. G**) increased also, trying to counteract further influx of 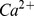. However, the increase in the 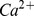 voltage dependent channel current (**Figure 8. D**) was substantially more marked, resulting in a net raise in the intracellular 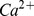 concentration.

Both Ca^2+^/insulin secretion experiments and preliminary computational results supported the hypothesis that L-alanine insulinotropic properties could be, at least in part, ascribed to it being co-transported with 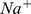. The model inputs are constituted by 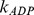 and 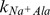
whereas the output is given by the 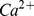 concentration. In order to validate our model with D-glucose or L-alanine insulin secretion dose-response curves, we made the following simplifying assumptions:

- Insulin secretion increases linearly with the bulk intracellular 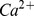 concentration [1],[51], (2);
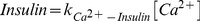
(2)

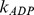
increases linearly with either D-glucose concentration or L-alanine concentration when they are administered separately. **Figure 3. A–B** demonstrates a quasi-linear relationship between D-glucose or L-alanine concentrations and their correspondent uptake. 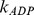 is the kinetic constant for metabolism-driven 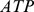 production and it is thus proportional to the nutrients uptake, (3);

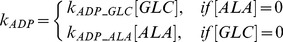
(3)

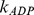
for 10 mmol /l L-alanine is equal to 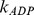 for 16.7 mmol/l D-glucose. This follows from the experimental 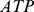 concentration corresponding to the highest L-alanine concentration administered (10 mmol/l) being comparable to 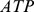 levels following 16.7 mmol/l D-glucose. (**Figure 3. C**), (4);

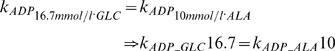
(4)
- The constant 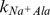 increases linearly with the concentration of L-alanine present in the buffer, (5).

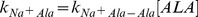
(5)We used a least-squared optimization criterion to fit the computational steady state intracellular 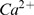 concentrations to the experimental insulin secretion observations. The fitting was performed using 11 observations in total taken from **Figure 2. A–B**: 1 with neither D-glucose nor L-alanine, 5 with increasing concentrations of D-glucose only and 5 with increasing concentrations of L-alanine only. We employed the MATLAB routine *fminsearch* to find 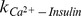, 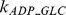 and 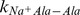
which minimise the objective function 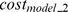 given by the squared difference between experimental insulin secretion data and simulated steady state 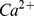 concentrations (6):
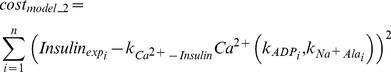
(6)


Where 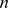 represents the number of experimental points, 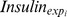
represents one of the insulin observations, the constant 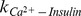 scales the simulated 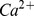 output to the 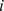 experimental insulin secretion values and 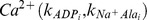 is the simulated mean steady state 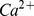 value with 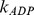 and 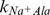 as per assumptions (ii)-(iv).

**Figure 9:**
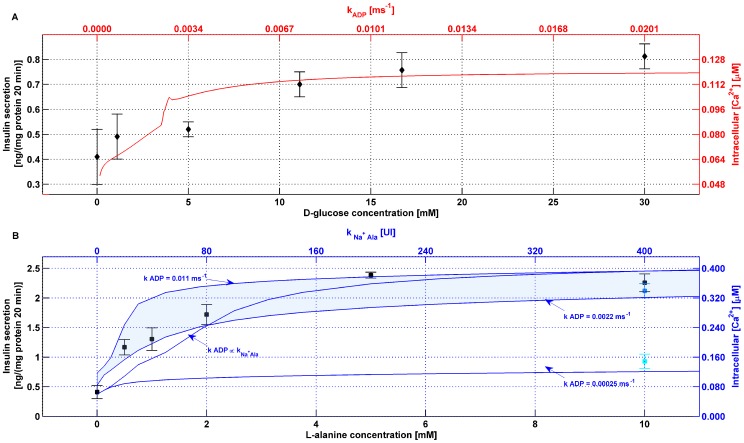
Comparison of simulated mean 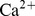 levels and experimental insulin secretion dose-response curves. **A.** Simulated steady state 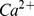 concentrations as a function of 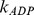 (top x axis and right y axis, in red) are overlaid onto the experimental D-glucose insulin secretion dose-response curve (bottom x axis and left y axis, in black). The top x axis was scaled to the bottom x axis with 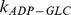 and the right y axis was scaled to the left y axis with 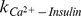, both found with the fitting. **B.** Simulated steady state 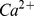 concentrations as a function of 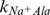 (top x axis and right y axis, in blue) are overlaid to the experimental L-alanine insulin secretion dose response curve (bottom x axis and left y axis, in black). The top x axis was scaled to the bottom x axis with 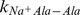 and the right y axis was scaled to the left y axis with 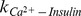, both found with the fitting. The line annotated 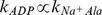 shows the simulated steady state 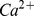 concentration if 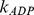 and L-alanine concentration follow assumption (ii). The patch delimited by the lines annotated 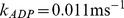 and 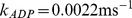, correspondent to simulated steady state value from 10 mmol/l L-alanine (or 16.7 mmol/l D-glucose) and a 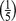 of it, shows that even a large variation in 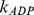 has only a modest effect on 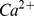. Administration of 10 mmol/ AIB with or without supplementation with 16.7 mmol/l D-glucose can be simulated by setting 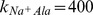 (same value as 10 mmol/l L-alanine) in both cases and 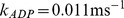 corresponding to 16.7 mmol/l D-glucose-derived ATP production (blue square) or 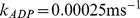 corresponding to virtually no metabolism-derived ATP production (cyan square), respectively. All simulations were performed using standard parameter values enumerated in **Table S2** except for 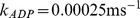 and 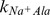 that assumed the values herein specified.

**Figure 9. A** shows the simulated steady state 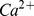 concentration as a function of 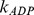 (top x axis and right y axis, in red) overlaid to the experimental D-glucose insulin secretion dose response curve (bottom x axis and left y axis, in black). The top x axis was scaled to the bottom x axis with 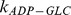 and the right y axis was scaled to the left y axis with 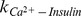, both found with the fitting.

Analogously, **Figure 9. B** shows the simulated steady state 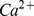 concentration as a function of 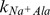 (top x axis and right y axis, in blue) overlaid to the experimental L-alanine insulin secretion dose response curve (bottom x axis and left y axis, in black). The top x axis was scaled to the bottom x axis with 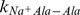 and the right y axis was scaled to the left y axis with 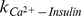, both found with the fitting.

The line annotated 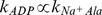 shows the simulated steady state 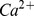 concentration if 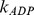 and L-alanine concentration are as laid out in assumption (ii).

The patch delimited by the lines annotated 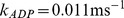 and 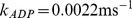, correspondent to simulated steady state value from 10 mmol/l L-alanine (or 16.7 mmol/l D-glucose) and a 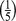
of it, shows that even a large variation in 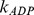 has only a modest effect on 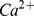.

The parameterised model was utilised to predict 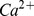 levels in other experimental conditions tested, such as administration of AIB. AIB is a non-metabolizable L-alanine analogue and thus does not lead to ATP production. However, AIB shares the same Na^+^ co-transport mechanism of L-alanine. Therefore, the administration of 10 mmol/ AIB with or without supplementation with 16.7 mmol/l D-glucose can be simulated by setting 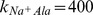 (same value as 10 mmol/l L-alanine) in both cases and 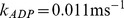
corresponding to 16.7 mmol/l D-glucose-derived ATP production (blue square) or 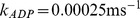 corresponding to virtually no metabolism-derived ATP production (cyan square), respectively (**Figure 9. B**). Note that experimental insulin secretion value following 10 mmol/l L-alanine only challenge is comparable to the value achieved when equimolar amount of AIB were supplemented with 16.7 mmol/l D-glucose.

The model is able to predict Ca^2+^ levels when D-glucose only, L-alanine only and D-glucose plus AIB, are administered. However, experimental data in **Figure 6** indicate that Ca^2+^ content is not the only player in insulin secretion when D-glucose and L-alanine are administered together. This finding suggests that the amplifying pathway, through ATP levels and possibly the putative messengers identified by the simulation of model 1, may markedly modulate GSIS in the presence of L-alanine. Therefore, currently the model is not able to predict correctly insulin secretion output when both stimuli are present together. A quantitative description for ATP-Ca^2+^ interaction as well as further characterization of the mechanism underlying the amplifying pathway are needed to overcome this limitation.

## Discussion

In this study, we built a comprehensive model of the role played by D-glucose and the amino acid L-alanine in promoting insulin secretion both independently and in combination to modulate GSIS *in vitro*. Previous experimental studies [5],[6],[8],[10],[53] have focussed on how L-alanine could enhance GSIS rather than considering the insulinotropic properties of the amino acid itself. Most GSIS mathematical models available in the literature focus on either metabolism or Ca^2+^ handling and none of these include possible mechanisms of action for amino acids. L-alanine is co-transported with Na^+^ ions into pancreatic β-cells where it is then converted to pyruvate and therefore can lead to insulin secretion by both directly depolarizing the plasma membrane and by oxidative metabolism.

We studied this using two separate models: a core metabolic model leading to ATP production (model 1) (**Figure 1. A**) and a Ca^2+^ handling model described by voltage-gated currents (model 2) (**Figure 1. B**).

We validated the models against *in vitro* observations on the BRIN-BD11 cell line using the workflow illustrated in **Figure 1. C**.

Total intracellular ATP observations exhibited a dose dependent response with respect to D-glucose which was further boosted by addition of 10 mmol/l L-alanine. Key kinetic parameters (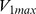, 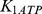, 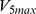
_,_ 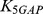, 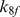, 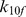, 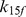, 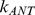, 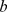 and 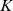) were fitted using a least squared criterion to make the model match ATP experimental results.

Simulations performed on the parameterized model 1 demonstrated a substantial increase in tzhe steady state concentration of L-glutamate when L-alanine influx was applied and even more strikingly if it was applied in conjunction with D-glucose influx (**Figure 5. A–B**). L-glutamate has been object of intense investigations and has been proposed to be a stimulation-secretion coupling factor in the amplifying pathway of insulin secretion [13],[54]–[56], although this hypothesis is controversial [57]. In order to test whether L-alanine could exploit its insulinotropic effects for both AASIS and GSIS through L-glutamate, we induced “artificially” an increase in the intracellular concentration of L-glutamate and we performed insulin secretion assays in parallel. A raise in intracellular L-glutamate concentration comparable in content to the increase induced by 10 mmol/l L-alanine at stimulatory concentration of D-glucose (16.7 mmol/l) was attained by using an L-glutamate precursor (5 mmol/l of di-methyl-glutamate). However, the corresponding insulin secretion was slightly but significantly decreased with respect to control (16.7 mmol/l D-glucose only with no other addition) without affecting cell viability (latter result not shown), (**Figure 5. C**). These results do not support the hypothesis that L-alanine impacts on insulin secretion solely via an increase in L-glutamate. However, the rise in L-glutamate may not be casually connected to L-alanine challenge. Our experiment focussed on clarifying the effect that L-glutamate generation may have *per se* in promoting insulin secretion. However, it remains plausible that L-glutamate plays an important coupling role in pancreatic β-cells, but other components may be necessary. It would be interesting to investigate whether the L-alanine-induced raise in Ca^2+^ levels had an effect on L-glutamate-mediated insulin secretion since it has been reported that L-glutamate failed to induce insulin secretion at basal intracellular Ca^2+^ concentration [13]. Furthermore, L-alanine-induced L-glutamate generation may be coupled to production of other products that we failed to reproduce in our experiments, which may be essential for the full development of L-glutamate-regulated insulin secretion. Interestingly, a recently published study [58] has shown, using high resolution immunocytochemistry techniques, that it is neither the increase in cytosolic nor intra-vesicular L-glutamate concentration that is responsible for the amplification of insulin secretion, but rather the flux of L-glutamate through the secretory granules that modulates both the pH and membrane potential which in turn shape the insulin response. However, the vast amount of conflicting data in literature, the very variable experimental conditions, numerous models employed, and the complexity of the pathways involved prevent a clear-cut interpretation. Therefore, future work should attempt to dissect the role of intracellular L-glutamate and potential cofactors in insulin secretion.

To gain further insights into what extent insulin secretory responses can be modulated by electrogenic Na^+^ co-transport, L-alanine was replaced in KRBB (with or without D-glucose supplementation) with equimolar amounts of α-aminoisobutyric acid (AIB). AIB is an L-alanine non metabolizable analogue, thus it shares the same co-transport mechanism of the amino acids without the capability of being oxidised and hence producing ATP. The latter finding is consistent with a previous study [17] where ouabain, a Na^+^ pump blocker, was used to demonstrate that L-alanine promotion of Na^+^ influx strongly affects insulin secretion. Moreover, it was demonstrated that replacing extracellular Na^+^ ions with equimolar concentration of N-methyl-D-glutamine (NMDG) in KRBB was effective in abolishing the effects of AIB and L-alanine electrical activity on elevation of intracellular Ca^2+^ [59] and insulin secretion, while GSIS was unaffected [17]. Interestingly, adding the mitochondrial poison oligomycin to L-alanine (alone or supplemented with D-glucose) and consequently almost abolishing the ATP output or replacing it with AIB led to a substantial and comparable decrease in insulin release.

The analysis of our model simulations indicates that both high intracellular ATP and Ca^2+^ concentrations are necessary to develop full insulin secretory responses, in agreement with previously published findings [1],[3],[11],[14],[17],[40],[52],[56] [60]. Importantly the model confirmed that Na^+^ co-transport acts synergistically with membrane depolarization and thus K^+^_ATP_ channel independent mechanisms of stimulation of Ca^2+^ levels in the β-cell that are essential for promotion of insulin secretion. Furthermore, both models combined suggest that the potent insulinotropic stimulus D-glucose plus L-alanine generates production of an, as yet, unidentified messenger involved in the amplifying pathway of insulin secretion.

Collectively experimental data and simulation results suggest that L-alanine is particularly effective in inducing insulin secretion through three independent and complimentary mechanisms of action. Firstly, L-alanine is metabolized at high rates [6] and, thus, can lead to a conspicuous ATP production resulting in insulin secretion through K^+^_ATP_ dependent triggering pathway. Simulations carried out on model 1 suggest that L-alanine is also the source of metabolically derived putative stimulus-secretion messengers in insulin secretion, such as L-glutamate and citrate (latter result not shown), which could account for the full development of insulin secretion through the amplifying pathway. Moreover, L-alanine / Na^+^ co-transport mediated depolarization accounts for a substantial proportion of L-alanine stimulated insulin secretion.

However, experimental data indicate that Ca^2+^ content is not the only player in insulin secretion when D-glucose and L-alanine are administered together (**Figure 6**).

In summary, L-alanine could be exploited for future studies of the mechanism(s) of powerful insulinotropic agents, as its action is robust, reliable, and controllable, contrarily from most commercially available drugs that lack coupling between metabolism and electrical activity.

The current model has multiple limitations. However, it is the only currently available model that accounts for amino acids-dependent insulin secretion mechanisms. Limitations of the current model includes the fact that model 1 in kinetic rates are either modelled using mass action or Michaelis-Menten functional expressions and do not take into account neither cooperativity nor other regulatory activities, like the Ca^2+^ effects on the mitochondrial dehydrogenases. However, the model is able to capture the essential behaviour of the experimental data, suggesting that the principal pathways involved are modelled with sufficient detail.

Furthermore, details of Na^+^ co-transport mechanism are still unknown and more comprehensive experimental data are needed to develop more informed expressions for the mathematical model 2 to be tested.

Our description of the β-cell’s core metabolic processes and Ca^2+^ handling could enable testing of metabolic hypotheses and drug actions and constitute a valuable tool in planning targeted experiments. Furthermore, this study could constitute a starting point for a future refinement of individual steps generating mitochondrial derived coupling factors and Ca^2+^ handling, thus allowing for future integration of other biochemical pathways of interest.

## Materials and Methods

### Experimental Methods

#### Reagents

RPMI-1640 culture media, foetal bovine serum (FBS) and plastic were purchased from Gibco (Glasgow, UK). All other chemicals were obtained from Sigma-Aldrich Chemical (Poole, Dorset, UK) unless stated otherwise.

#### Culture of BRIN-BD11 cells

BRIN-BD11 cells were grown and maintained in RPMI-1640 tissue culture medium with 10% (v/v) foetal bovine serum, 0.1% antibiotics (100 units/ml penicillin and 100 µg/ml streptomycin), 2 mmol/l L-glutamine and 11.1 mmol/l D-glucose, pH 7.4. BRIN-BD11 cells were grown and maintained at 37°C in a humidified atmosphere of 5% CO_2_ and 95% air using a Forma Scientific incubator (Marietta, OH, USA). Cells were cultured in 50–70 ml of RPMI-1640 tissue culture medium in T175cm^2^ sterile tissue culture flasks (Greiner) and passaged every 2–3 days by mechanical detachment with 0.025% (w/v) trypsin/EDTA solution. The passage number of cells used in this study was in the range 18–30.

The general experimental workflow is illustrated in **Figure 1.C**.

Briefly, BRIN-BD11 cells were seeded into either 24-well plates or T75cm^2^/T175cm^2^ flasks depending on the parameter to be assayed and were allowed to adhere over night before experiments. The following day, cells were washed with calcium and magnesium free phosphate buffer saline (PBS) and pre-incubated at 37°C for 40 min in Krebs Ringer bicarbonate buffer (KRBB) (115 mmol/l NaCl, 4.7 mmol/l KCl, 1.28 mmol/l CaCl_2_, 1.2 mmol/l KH_2_PO_4_, 1.2 mmol/l MgSO_4_ ˙7H_2_0, 10 mmol/l NaHCO_3_, 1 g/l BSA, pH 7.4) supplemented with 1.1 mmol/l D-glucose. Supernatant was carefully removed and discarded before cells were washed with PBS.

This was followed by an incubation period of 20 min in KRBB as described above but supplemented with either D-glucose only (1.1, 5, 16.7, 30 mmol/l) or L-alanine only (0.5, 1, 2, 5, 10 mmol/l) or various combinations of D-glucose and L-alanine.

After incubation, supernatant was removed, centrifuged (400 **g** for 5 min at 4°C) and stored at −20°C for later determination of insulin and metabolites (D-glucose, L-alanine and L-lactate). Intracellular Ca^2+^ concentration was assessed by a cell-based experiment performed with flow cytometric techniques. Cells were also analysed for their respective ATP and L-glutamate content (lysates were stored at −80°C).

All the results (except cell viability measurements and intracellular Ca^2+^ concentrations) were normalized with respect to the cell protein content.

Each experiment was carried out on at least three independent cultures of BRIN-BD11 cells.

#### Cell viability measurements

Cell viability was assessed by neutral red uptake assay which provides a quantitative estimation of the number of viable cells based on their ability to incorporate and bind the dye neutral red in lysosomes.

BRIN-BD11 cells were seeded in 24 well plates (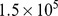 per ml) and let to adhere overnight.

BRIN-BD11 cells were incubated for 1 h in KRBB supplemented with different concentrations of either D-glucose or L-alanine or various combinations of both in the presence of DMSO-dissolved neutral red (100 µg/ml). Subsequently, cells were washed twice with PBS and the dye was extracted by disruption with acid ethanol (alcohol/glacial acetic acid, 50∶1 v/v). An aliquot (100 µl) of the resulting solutions was transferred to a 96-well plate and absorbance at 540 nm was recorded using a microplate spectrophotometer (Molecular Devices SpectraMax Plus 384, Sunnyvale, CA, USA).

#### Insulin Secretion

BRIN-BD11 cells (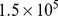 per ml) were seeded in 24-well plates and allowed to adhere overnight. Cells were then starved and stimulated as described above and aliquots of incubation KRBB were stored at −20°C and subsequently assayed for insulin using a Mercordia (Uppsala, Sweden) ultrasensitive rat insulin enzyme-linked immune-absorbent assay (ELISA) according to the manufacturer’s instructions. Absorbance readings were performed using a microplate spectrophotometer (Molecular Devices SpectraMax Plus 384, Sunnyvale, CA, USA).

It was ensured that cell density and insulin content were not significantly different under any of the incubation conditions [6].

BRIN-BD11 cells were lysed by addition of 150 µl of 1X RIPA buffer to each well and incubation for 1 h. Lysates were then transferred to Eppendorfs tubes, centrifuged at 14000 **g** for 15 min at 4°C. Supernatant were then frozen at −20°C for later protein analysis.

#### Total intracellular ATP concentration

BRIN-BD11 cells were seeded in a T175cm^2^ sterile tissue culture flask (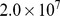
cells per flask) and allowed to adhere overnight. After starvation and stimulation as described above, cells were trypsinized and re-suspended in KRBB. The cell suspension was spun at 400 **g** for 5 min at 4°C, supernatant was removed and pellets were shock frozen in liquid N_2_ before being stored at −80°C. Cells were lysed with a suitable lysis buffer and the ATP released was quantified using a commercial available kit (ATP Bioluminescence Assay Kit HS II, Roche, Mannheim, Germany) according to instructions of the manufacturer and results were recorded using a luminometer (Turner Biosystems, Modulus™ Microplate Multimode Reader, Sunnyvale, CA). The assay is based on the ATP dependency of the light emitting luciferase-catalysed oxidation of luciferin for accurate measurement of extremely low concentration of ATP. An aliquot of cells lysates was stored at −80°C for later determination of proteins content.

#### Intracellular Ca^2+^ concentration

BRIN-BD11 cells were seeded in a T75cm^2^ flask (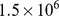 cells per flask) and allowed to adhere overnight. Cells were washed with PBS, incubated with complete media loaded with 1 µM of the probe Fluo-4 AM (Molecular Probes, Invitrogen Life Technologies, CA, USA) for 1 h, washed twice with PBS, trypsinized, re-suspended in KRBB supplemented with 1.1 mmol/L D-glucose and starved for 40 min. Subsequently, cells were briefly centrifuged (400 **g** for 5 min at 4°C), washed with PBS, briefly centrifuged again and incubated for further 20 minutes in KRBB supplemented with test stimuli. Intracellular Ca^2+^ concentrations were determined using an Accuri 6 flow cytometer (Accuri, Ann Arbor, MI, USA). Fluo-4 AM dye was excited by a solid-state blue laser (488 nm), 50000 events per sample were acquired at a slow flow rate (14 µl/min, 10 µm core) and the fluorescent emissions were detected at 530/30 band pass filter (FL1). Data were acquired and analysed with BD Accuri CFlow® software and results are presented as percentage of control (KRBB with no additional stimuli).

#### Enzymatic determinations of metabolites

D-glucose consumption, L-alanine consumption and L-lactate production assays were carried out in 24 well plates (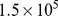 cells per ml). After starvation and stimulation as described previously, aliquots of KRBB used to stimulate the cells were taken for analysis at 0 min and 20 min, centrifuged at 400 **g** for 5 min at 4°C.

D-Glucose and L-Alanine consumption were determined using quantitative enzymatic colorimetric assays, LiquiColor (Stanbio, Boerne, TX, USA) and (BioVision, Miltipitas California, USA), respectively and YSI 7100 Multiparameter Bioanalytical System (Life Sciences, Fleet, UK). L-lactate production was measured using a lactate oxidase based assay kit supplied by BioVision.

Analogously to insulin secretion assays, cell proteins were incubated for 1 h with 150 µl of 1 X RIPA lysis reagent. Cells lysates were then removed to fresh ice-cold micro-centrifuge tubes and centrifuged at 14000 **g** for 15 min at 4°C. The supernatant fraction was transferred to a fresh tube and stored at −20°C for later analysis.

For L-glutamate determination, BRIN-BD11 cells were seeded in a T175cm^2^ flask (2.0×10^7^ cells), starved and stimulated as described previously. Subsequently, supernatant was removed and discarded, cells were washed twice with ice-cold PBS, lysed and total intracellular L-glutamate was determined according to the manufacturer’s instructions using a colorimetric method (BioVision). An aliquot of lysates was stored at −80°C for later protein determination.

#### Protein determination

Cellular protein content was quantified using a BCA (bicinchoninic acid) protein assay kit (Pierce, Rockford, IL, USA; kit no.23225), which utilizes a modification of the Biuret reaction.

**Cell size measurement**

BRIN-BD11 cell size was determined by using two independent approaches: (i) an indirect computer-based approach and (ii) direct measurement in suspension.

- BRIN-BD11 cells were washed with PBS twice, trypsinized, and removed in KRBB supplemented with 11.1 mmol/l D-glucose and 0.005% DMSO-dissolved neutral red. BRIN-BD11 cells were seeded at low density (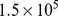 per ml) on a haemocytometer and allowed to adhere for 6 hours. Subsequently, BRIN-BD11 cells were washed thoroughly with PBS and high-power images (400 X magnification) were obtained using a BX51 Olympus microscope (Olympus Life Science Microscopes, Munich, Germany) and an Olympus DP71 camera. Subsequently, images were imported in MATLAB, processed (threshold and thinning) and cell size was quantified by proportion between pixel numbers and known dimension of the haemocytometer square.
- BRIN-BD11 were detached by trypsinization, re-suspended in complete media and removed into an Eppendorf at low density (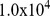 per ml) and subsequently their average diameter was measured with a 60 µm scepter sensor (catalogue number: PHCC60050, Millipore) mounted on an impedance-based handheld automated cell counter (Scepter™ 2.0, Millipore). Data were captured, manually gated to exclude cell debris and analysed using the Scepter™ Software Pro.

**Statistical analysis**

The results are presented as mean ± SD (standard deviation). Groups of data were compared using an unpaired Student’s t test or one-way ANOVA, where appropriate. The level of significance was set at P <0.05.

### Mathematical Modelling

We considered the following mathematical models focussing on:

1. L-alanine trigger of insulin secretion and how it enhances GSIS through stimulation of oxidative metabolism;
2. The effect of direct membrane depolarization caused by L-alanine / Na^+^-co-transport on triggering Ca^2+^ influx.

We separated the insulin secretion pathway into two main modules: (i) core metabolic processes leading to ATP production with either D-glucose and/or L-alanine as input (**Figure 1. A**) and (ii) electrophysiological downstream events with metabolism-derived ATP as input and Ca^2+^ influx as output (**Figure 1. B**).

The model incorporates the salient features of metabolic processes and electrophysiology and their interactions without explicitly modelling insulin secretion.

Both models were implemented, analysed and numerically simulated in MATLAB using the built in solver *ode15s*.

Each model is described in detail separately below.

#### Core metabolic processes model

The essential components of energy metabolism responsible for ATP production following D-glucose and/or L-alanine input are schematized in **Figure 1. A**.

We used a simple core metabolic model that includes glycolysis, TCA cycle, L-alanine-related reactions, respiratory chain and ATP synthesis machinery based on previous work by Nielsen [33] for the glycolytic pathway and Mazat [46] for the mitochondrial metabolism.

Enzymatic reactions are modelled using either mass action or Michaelis-Menten kinetics and they are based on the original studies [33],[46] and adopted with minor modifications to represent pancreatic β-cells.

Five additional reactions (solid blue lines in **Figure 1. A**) were included in order to account for metabolism of L-alanine:

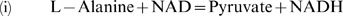

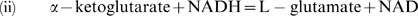

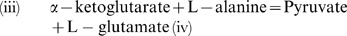

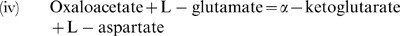

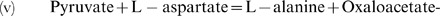
For simplicity, L-alanine-specific reactions are modelled as reversible mass-action kinetics as in [46]:

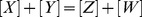
(7)

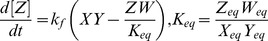
(8)Where 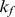
represents the rate of the forward reaction, 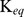 the equilibrium constant of the reaction whereas 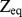, 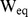 and 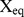, 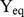 are the equilibrium concentrations of products and reactants, respectively.

Only one compartment is taken into account: cytosolic species are instantaneously carried across the mitochondrial membrane and their relative transporters are assumed to work in a region far from saturation.

The model describes the dynamics of 18 state variables for the glucose 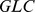, fructose 6-phosphate 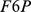, fructose 1,6-phosphate 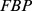, glyceraldehyde 3-phosphate 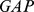, 1,3-biphosphoglycerate 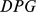, phosphoenol pyruvate 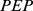, pyruvate 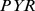, L-lactate 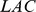, acetyl co-enzyme A 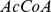, oxaloacetate 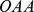, citrate 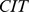, α-ketoglutarate 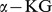, L-glutamate 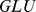, L-aspartate 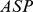, L-alanine 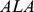 and the mitochondrial membrane potential (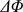). The resulting ordinary differential equation system is presented below:
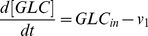
(9)

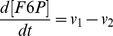
(10)

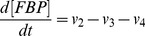
(11)

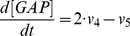
(12)

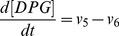
(13)

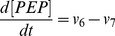
(14)

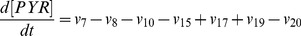
(15)

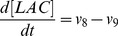
(16)

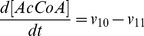
(17)

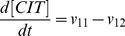
(18)

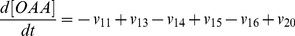
(19)

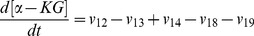
(20)

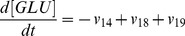
(21)

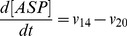
(22)

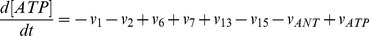
(23)

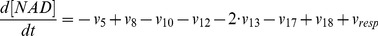
(24)

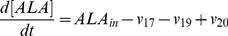
(25)

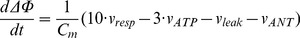
(26)


The complete list of the reactions included in the model and the corresponding rate equations (27–51) can be found in **Table 2** whereas initial conditions are enumerated in the first section of **Table S1** along with references to the original studies.

**Table 2:** Mathematical model of core metabolic processes in pancreatic β-cells: reactions list.

Reaction	Rate expression	Eq.
1. GLC+ATP→F6P+ADP	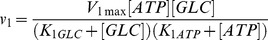	(27)
2. F6P+ATP→FBP+ADP	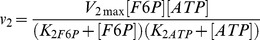	(28)
3. FBP→out	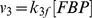	(29)
4. FBP = 2 GAP	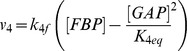	(30)
5. GAP+NAD→DPG+NADH	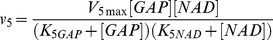	(31)
6.DPG+ADP→PEP+ATP	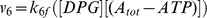	(32)
7. PEP+ADP→PYR+ATP	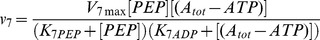	(33)
8. PYR+NADH→LAC+NAD^+^	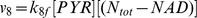	(34)
9. LAC→out	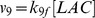	(35)
10. PYR+NAD^+^→AcCoA+NADH	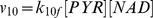	(36)
11. AcCoA+OAA→CTT	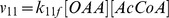	(37)
12. CTT+NAD^+^→α−KG+NADH	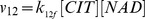	(38)
13. α−KG+2.NAD^+^+ADP→OAA+2.NADH+ATP	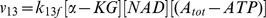	(39)
14. OAA+GLU = α−KG+ASP	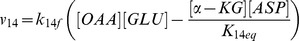	(40)
15. PYR+ATP→OAA+ADP	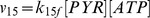	(41)
16. OAA→out	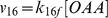	(42)
17. ALA+NAD^+^ = PYR+NADH	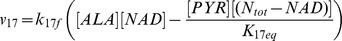	(43)
18. α−KG+NADH = GLU+NAD^+^	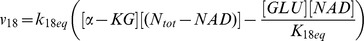	(44)
19. ALA+α−KG = PYR+GLU	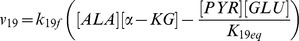	(45)
20. PYR+ASP = ALA+OAA	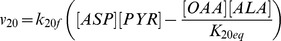	(46)
21. ATP→ADP	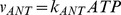	(47)
22. H^+^_e_→H^+^	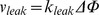	(48)
23. NADH+ 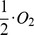 11.H^+^→NAD^+^+ H_2_O+10.H^+^_e_	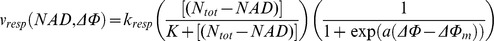	(49)
24. ADP+Pi+3.H^+^_e_ = ATP+H_2_O+3.H^+^	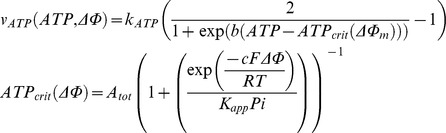	(50) (51)

**Abbreviations of substrates**: 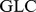, D-glucose; 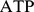, adenosine triphosphate; 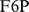, fructose-6-phosphate; 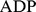, adenosine diphosphate; 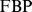, fructose-1,6-biphopshate; 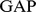, glyceraldehyde 3-phosphate; 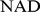 and 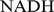, nicotinamide adenine dinucleotides; 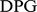, 1,3-biphosphoglycerate; 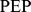, phosphoenol pyruvate; 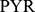, pyruvate; 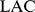, L-lactate; 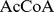, acetyl coenzyme A; 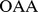, oxaloacetate; 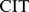, citrate; 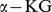, α-ketoglutarate; 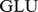, L-glutamate; 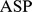, L-aspartate; 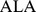, L-alanine; 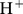, hydrogen ion; 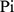, inorganic phosphate.

**Calcium handling model**

We are interested in computing whether an additional Na^+^ influx brought about by L-alanine co-transport can significantly increase the mean intracellular Ca^2+^ concentration at the steady state, possibly accounting for a substantial raise in insulin secretion.

Membrane processes in β-cell are modelled according to the Hodgkin-Huxley theory of membrane electrical activity in which the rate of change of the plasma membrane potential 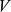
is given by the current balance equation (52), [43]:
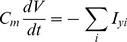
(52)Where 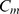
is the membrane capacitance and 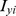
is the ionic current due to ion 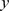
across the channel 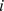 described by (53), [43]:



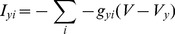
(53)

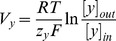
(54)With 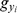 the cell conductance, 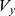 is the reversal Nernst equilibrium potential, 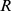 the gas constant, 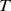 the absolute temperature, 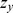 the ionic valence and 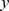 the concentration of the ion on the respective side of the plasma membrane [43].

Our model is based on the work of Fridlyand [43] and uses ATP derived from metabolism as input, as shown in **Figure 1. B**. The original model includes equations for the Endoplasmatic Reticulum (ER) Ca^2+^ sequestration events that have been reported in literature and are believed to modulate the burst mechanism. However, since electrophysiological events in β-cells are primarily driven by the plasma membrane oscillator and to date there are no data indicating that Na^+^ co-transport might affect ER Ca^2+^ stores or the sarco(endo)plasmic reticulum Ca^2+^ ATPase pumping rate, ER equations are not included in our model. Therefore, only one compartment was taken into account: we did not distinguish upon Ca^2+^ located in the cytosol or in the intracellular stores since our experimental data quantified only the total intracellular Ca^2+^ content. The model assumes that the extracellular space is constituted by a large volume, so that extracellular 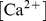, 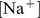 and 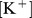 are treated as a thermodynamic bath and can be considered constant. Extracellular concentrations of Ca^2+^, Na^+^ and K^+^ are taken from [43] and their values are in good agreement with the composition of KRBB used for the experimental work.

Pancreatic β-cells are characterised by a range of different arrays of channels, however we only included the ones that are most relevant to our object of investigation. In particular, a delayed rectifying K^+^ current (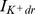), K^+^ ATP-dependent current (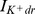), K^+^ Ca^2+^-activated current (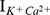), Ca^2+^ plasma membrane pump (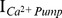), Ca^2+^ uniporter (voltage-dependent) current (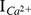), Na^+^ voltage-gated current (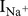), Na^+^/K^+^ pump current (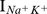), Na_+_/Ca^2+^ exchanger current (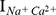) were included in this model. The mathematical formulations of these currents and Nernst potential equations (55–69) were adopted without major modifications from [43] and are listed in **Table 3**.

**Table 3:** Mathematical model of 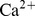 handling in pancreatic β-cells: Nernst potential and channels currents list.

Ion specie(s)	Nernst potential equation	Eq.
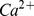	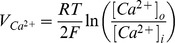	(55)
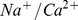	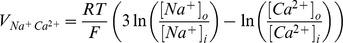	(56)
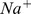	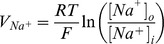	(57)
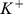	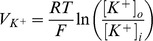	(58)

In order to account for L-alanine/Na^+^-co-transport, we introduced an additional Na^+^ current to model the increase in intracellular Na^+^ concentration that accompanies L-alanine uptake.

Although the detailed mechanism of L-alanine / Na^+^ symport is not currently known, a study carried out on BRIN-BD11 has provided evidence that the co-transport occurs through a Na^+^ tetrodotoxin (TTX)-insensitive channel [17]. Extracellular Na^+^ concentration is not the limiting factor for the transport as its level is one order of magnitude higher than the corresponding intracellular value. We model the Na^+^ current (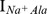) due to L-alanine/Na^+^ co-transport by re-adapting an expression previously used to model an inward Na^+^ current through a TTX-insensitive channel [39],[61]. We then use the constant 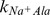 as an adjustable parameter to modulate the current flowing through this channel as a function of L-alanine concentration in the KRRB.
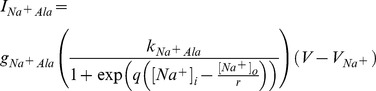
(69)


The dynamic changes in intracellular concentrations of 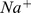 and 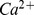 are explicitly modelled as well as the membrane potential (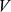), a voltage-dependent gating variable (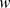) and intracellular 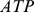 concentration. The resulting system of differential equations is given by:
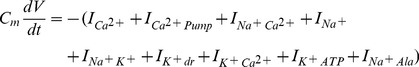
(70)

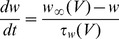
(71)

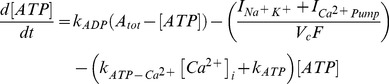
(72)

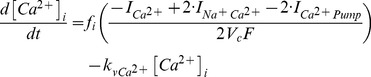
(73)

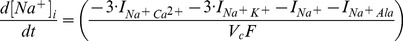
(74)


Initial conditions are listed in the first section of **Table S2**.

#### Parameter Values

Where possible the parameters used in the model come directly from the literature, are extrapolated from the literature, are re-calculated from other published models or are fitted to the experimental data described herein. The full list of parameters and their values are given in **Table S1** for the core metabolic processes model and in **Table S2** for the Ca^2+^ handling model.

## Supporting Information

Table S1 **Mathematical model of core metabolic processes in pancreatic β-cells: initial conditions and standard parameters list**.
(PDF)

Table S2 **Mathematical model of Ca^2+^ handling in pancreatic β-cells: initial conditions and standard parameters list.**
(PDF)
