# Supplementary material for: Mathematical Model of Metabolism and Electrophysiology of Amino Acid and Glucose Stimulated Insulin Secretion: In Vitro Validation Using a β-Cell Line
Source: PLoS One. 2013 Mar 8;8(3):e52611. doi: 10.1371/journal.pone.0052611 (PMC3592881; doi:10.1371/journal.pone.0052611)
Supplement: Table S1 — Mathematical model of core metabolic processes in pancreatic β-cells: initial conditions and standard parameters list. (PDF) [file pone.0052611.s001.pdf]

Table S1. Mathematical model of core metabolic processes in pancreatic β-cells: initial conditions and standard parameters list.

INITIAL CONDITION	DESCRIPTION	UNITS	VALUE	EQUATION	REFERENCE
GLC_0	$GLC(t=t_0)$	M	6.16×10^{-4}	9	[33]
$F6P_0$	$F6P(t=t_0)$	M	6.59×10^{-3}	10	[33]
FBP_0	$FBP(t=t_0)$	M	7.70×10^{-6}	11	[33]
GAP_0	$GAP(t=t_0)$	M	1.91×10^{-6}	12	[33]
DPG_0	$DPG(t=t_0)$	M	2.99×10^{-4}	13	[33]
PEP_0	$PEP(t=t_0)$	M	2.11×10^{-6}	14	[33]
PYR_0	$PYR(t=t_0)$	M	4.23×10^{-6}	15	[46]
LAC_0	$LAC(t=t_0)$	M	7.38×10^{-2}	16	[33]
$AcCoA_0$	$AcCoA(t=t_0)$	M	6.30×10^{-5}	17	[46]
CIT_0	$CIT(t=t_0)$	M	3.60×10^{-4}	18	[46]
OAA_0	$OAA(t=t_0)$	M	4.50×10^{-6}	19	[46]
$\sim -KG_0$	$\sim -KG(t=t_0)$	M	2.25×10^{-4}	20	[46]
GLU_0	$GLU(t=t_0)$	M	5.30×10^{-3}	21	[34]
ASP_0	$ASP(t=t_0)$	M	1.60×10^{-3}	22	[34]
ATP_0	$ATP(t=t_0)$	M	3.00×10^{-3}	23	[46]
NAD_0	$NAD(t=t_0)$	M	1.00×10^{-3}	24	[34]
ALA_0	$ALA(t=t_0)$	M	7.00×10^{-4}	25	[34]
$\Delta\Phi_0$	$\Delta\Phi(t=t_0)$	V	1.00×10^{-1}	26	[46]
**PARAMETER**	**DESCRIPTION**	**UNITS**	**VALUE**	**EQUATION**	**REFERENCE**
**Glycolysis**					
V_{1max}	Maximal rate of v_1	$M\ s^{-1}$	3.96×10^{-4}	27	Fitted

K_{1GLC}	M-M constant for GLC	M	1.00 x 10^{-4}	27	[33]
K_{1ATP}	M-M constant for ATP	M	6.00 x 10^{-3}	27	Fitted
$V_{2\max}$	Maximal rate of v_2	M s^{-1}	1.50 x 10^{-3}	28	[33]
K_{2F6P}	M-M constant for F6P for v_2	M	1.60 x 10^{-5}	28	Recalculated from [33]
K_{2ATP}	M-M constant for ATP for v_2	M	1.00 x 10^{-5}	28	[33]
k_{3f}	Rate of elimination of $FBP\,(v_3)$	s^{-1}	5.00 x10^{-2}	29	[33]
k_{4f}	Forward rate constant for v_4	s^{-1}	1.00	30	[33]
K_{4eq}	Equilibrium constant for v_4	M^{-1}	2.00 x 10^{-2}	30	[33]
$V_{5\max}$	Maximal rate of v_5	M s^{-1}	5.00 x 10^{-3}	31	Fitted
K_{5GAP}	M-M constant for GAP for v_5	M	4.50 x 10^{-2}	31	Fitted
K_{5NAD}	M-M constant for NAD for v_5	M	1.00 x 10^{-3}	31	[33]
k_{6f}	Forward rate constant for v_6	M^{-1} s^{-1}	1.00 x 10^{3}	32	[33]
$V_{7\max}$	Maximal rate of v_7	M s^{-1}	1.00 x 10^{-2}	33	[33]
K_{7PEP}	M-M constant for PEP for v_7	M	2.00 x 10^{-4}	33	[33]
K_{7ADP}	M-M constant for ADP for v_7	M	3.00 x 10^{-4}	33	[33]

k_{8f}	Forward rate constant for v_8	$M^{-1}\ s^{-1}$	325	34	Fitted
k_{9f}	Rate constant for LAC disposal (v_9)	s^{-1}	1	35	Estimated
**TCA cycle**					
k_{10f}	Forward rate constant for v_{10}	$M^{-1}s^{-1}$	35	36	Fitted
k_{11f}	Forward rate constant for v_{11}	$M^{-1}s^{-1}$	57142	37	[46]
k_{12f}	Forward rate constant for v_{12}	$M^{-1}s^{-1}$	53	38	[46]
k_{13f}	Forward rate constant for v_{13}	$M^{-2}s^{-1}$	82361	39	[46]
k_{14f}	Forward rate constant for v_{14}	$M^{-1}\ s^{-1}$	72	40	Recalculated from [46]
K_{14eq}	Equilibrium constant for v_{14}	Ul	10.19	40	Recalculated from [46]
k_{15f}	Forward rate constant for v_{15}	$M^{-1}\ s^{-1}$	12	41	Fitted
k_{16f}	Rate of elimination of OAA (v_{16})	s^{-1}	3.60	42	[46]
**Alanine-related reactions**					
k_{17f}	Forward rate constant for v_{17}	$M^{-1}\ s^{-1}$	70	43	Estimated
K_{17eq}	Equilibrium constant for v_{17}	Ul	31	43	Estimated
k_{18f}	Forward rate constant for v_{18}	$M^{-1}\ s^{-1}$	1.38	44	Estimated

K_{18eq}	Equilibrium constant for v_{18}	Ul	163.08	44	Estimated
k_{19f}	Forward rate constant for v_{19}	M^{-1} s^{-1}	1.20	45	Estimated
K_{19eq}	Equilibrium constant for v_{19}	Ul	77	45	Estimated
k_{20f}	Forward rate constant for v_{20}	M^{-1} s^{-1}	155	46	Estimated
K_{20eq}	Equilibrium constant for v_{20}	Ul	22.30	46	Estimated
**Respiratory chain, ATP synthesis machinery & ATP translocation**					
k_{ANT}	Rate of anti-translocation	s^{-1}	9.53×10^{-2}	47	[46]
k_{leak}	Rate of proton leak	$M\ V^{-1}s^{-1}$	4.26×10^{-4}	48	[46]
C_m	Membrane Capacitance	$M\ V^{-1}$	6.75×10^{-6}	26	[46]
k_{resp}	Respiration kinetic constant	$M\ s^{-1}$	2.50×10^{-3}	49	[46]
K	M-M like constant for NADH	M	2.50×10^{-3}	49	[46]
a	Constant	Ul	100	49	[46]
$\Delta\Phi_m$	Threshold mitochondrial membrane potential	V	0.15	49	[46]
N_{tot}	Total nicotinamide adenine dinucleotide concentration	M	1.07×10^{-3}	34, 43, 44, 49	[46]

A_{tot}	Total adenosine di- and triphosphate concentration	M	4.16×10^{-3}	32,33, 39, 51	[46]
k_{ATP}	ATP kinetic constant	$M\ s^{-1}$	0.1319	50	[46]
b	ATPase constant	Ul	54	50	Fitted
K_{app}	Apparent equilibrium constant	Ul	4.40×10^{-6}	51	[46]
**Physical constants**					
R	Ideal gas constant	$J\ mol^{-1}\ K^{-1}$	8.31	51	-
T	Mammalian body temperature	K	310	51	-
F	Faraday's constant	$C\ mol^{-1}$	9.65×10^{4}	51	-
c	Constant	Ul	3.60	51	[46]

Ul, unitless, M-M, Michaelis-Menten.

If the D-glucose influx assumes values that exceed the maximum flux through reaction 1 ($GLC + ATP \rightarrow F6P + ADP$), a linear leak term can be used to allow D-glucose to reach a steady state.

References:

[33] Nielsen K, Sørensen PG, Hynne F, Busse HG (1998) Sustained oscillations in glycolysis: an experimental and theoretical study of chaotic and complex periodic behavior and of quenching of simple oscillations. Biophysical Chemistry 72: 49-62.

[34] Jiang N, Cox R, Hancock J (2007) A kinetic core model of the glucose-stimulated insulin secretion network of pancreatic β cells. Mammalian Genome 18: 508-520.

[46] Nazaret C, Heiske M, Thurley K, Mazat JP (2009) Mitochondrial energetic metabolism: a simplified model of TCA cycle with ATP production. J Theor Biol 258: 455-464.
