# Supplementary material for: Mathematical Model of Metabolism and Electrophysiology of Amino Acid and Glucose Stimulated Insulin Secretion: In Vitro Validation Using a β-Cell Line
Source: PLoS One. 2013 Mar 8;8(3):e52611. doi: 10.1371/journal.pone.0052611 (PMC3592881; doi:10.1371/journal.pone.0052611)
Supplement: Table S2 — Mathematical model of Ca2+ handling in pancreatic β-cells: initial conditions and standard parameters list. (PDF) [file pone.0052611.s002.pdf]

Table S2. Mathematical model of Ca^{2+} handling in pancreatic β-cells: initial conditions and standard parameters list.

INITIAL CONDITION	DESCRIPTION	UNITS	VALUE	EQUATION	REFERENCE
V_0	$V(t=t_0)$	mV	-60.9	70	[43]
w_0	$w(t=t_0)$	Ul	0.00123	71	[43]
$\left[Ca^{2+}\right]_0$	$\left[Ca^{2+}\right](t=t_0)$	µM	0.085	73	[43]
$[ATP]_0$	$[ATP](t=t_0)$	µM	932	72	[43]
$\left[Na^{+}\right]_0$	$Na^{+}(t=t_0)$	µM	9858	74	[43]
**PARAMETER**	**DESCRIPTION**	**UNITS**	**VALUE**	**EQUATION**	**REFERENCE**
$\left[Ca^{2+}\right]_o$	Extracellular [Ca^{2+}]	mM	2.5	55-56	[43]
$\left[Na^{+}\right]_o$	Extracellular [Na^{+}]	mM	140	56-57, 69	[43]
$\left[K^{+}\right]_o$	Extracellular [K^{+}]	mM	8	58	[43]
$\left[K^{+}\right]_i$	Intracellular [K^{+}]	mM	132.4	58	[43]
$g_{Ca^{2+}}$	Maximum conductance	pS	770	59	[43]
$K_{Ca^{2+}h}$	Slope at half activation potential	mV	9.5	59	[43]
$V_{Ca^{2+}h}$	Half-activation potential	mV	-19	59	[43]
$P_{Ca^{2+}Pump}$	Maximum current through the pump	fA	2000	60	[43]
$K_{Ca^{2+}Pump}$	Half-maximal pump activity	µM	0.1	60	[43]
$g_{Na^{+}Ca^{2+}}$	Maximum conductance	pS	271	61	[43]
$K_{Na^{+}Ca^{2+}}$	Ca^{2+} affinity constant	µM	0.75	61	[43]

g_{Na^+}	Maximum conductance	pS	1200	62	[43]
V_{Na^+ha}	Half activation Na^+ potential	mV	104	62	[43]
S_{Na^+}	Slope at half maximum Na^+ potential	mV	8	62	[43]
$P_{Na^+K^+}$	Current coefficient	fA ms	600	63	[43]
d	Numerator coefficient 1	μM^{-4} ms^{-6}	9.4944 x 10^{-12}	63	[43, 50]*
e	Numerator coefficient 2	μM^{-1} ms^{-6}	0.1474	63	[43, 50]*
f	Denominator coefficient 1	μM^{-1} ms^{-5}	0.4177	63	[43, 50]*
g	Denominator coefficient 2	μM^{-2} ms^{-5}	2.3165 x 10^{-4}	63	[43, 50]*
h	Denominator coefficient 3	μM^{-1} ms^{-5}	5.3733 x 10^{-3}	63	[43, 50]*
h_1	Denominator coefficient 4	Ul	2.6087 x 10^{-3}	63	[43, 50]*
g_{K^+dr}	Maximal conductance	pS	3000	64	[43]
V_w	Half activation potential	mV	-14	65	[43]
S_w	Slope at half maximal potential	mV	7	65	[43]
V_z	Relaxation potential coefficient	mV	-75	66	[43]
a	Relaxation coefficient	mV	65	66	[43]
b	Relaxation coefficient	mV	20	66	[43]
c	Relaxation coefficient	ms	20	66	[43]
$g_{K^+Ca^{2+}}$	Maximal conductance	pS	130	67	[43]
$K_{K^+Ca^{2+}}$	Affinity constant	µM	0.1	67	[43]
g_{K^+ATP}	Maximal conductance	pS	24000	68	[43]
i	Numerator coefficient 1	Ul	0.08	68	[43]

l	Numerator coefficient 2	Ul	0.0264	68	[43]
m	Numerator coefficient 3	Ul	0.0242	68	[43]
n	Denominator coefficient 1	Ul	0.1650	68	[43]
o	Denominator coefficient 2	Ul	0.1350	68	[43]
p	Denominator coefficient 3	Ul	0.0500	68	[43]
K_{dd}	ADP dissociation constant	µM	17	68	[43]
K_{tt}	ATP dissociation constant	µM	1	68	[43]
K_{td}	ADP-ATP dissociation constant	µM	26	68	[43]
$g_{Na^{+}Ala}$	conductance	pS	1	69	[17, 39, 61]
$k_{Na^{+}Ala}$	L-alanine dependence	Ul	Adj	69	[17, 39, 61]
q	coefficient	Ul	5.00 x 10^{-3}	69	[17, 39, 61]
r	coefficient	Ul	28	69	[17, 39, 61]
$k_{vCa^{2+}}$	Ca^{2+} sequestration rate	ms^{-1}	0.0005	73	Adapted from [43]
k_{ADP}	ATP production rate	ms^{-1}	Adj	72	[43]
$k_{ATP-Ca^{2+}}$	Ca^{2+}-dependent ATP consumption rate	$\mu M^{-1}ms^{-1}$	0.00005	72	[43]
k_{ATP}	ATP consumption rate	ms^{-1}	0.00005	72	[43]
A_{tot}	Total intracellular nucleotide	µM	4000	63, 68, 72	[43]
f_i	Fraction of cytoplasmatic free Ca^{2+}	Ul	0.01	73	[43]
C_m	Membrane capacitance	fF	6158	70	[43]
R	Ideal gas constant	J mol^{-1} K^{-1}	8.31	55-58, 63	-
T	Mammalian body temperature	K	310	55-58, 63	-
V_c	Cytosolic volume	pL	0.764	72-74	[43]

F	Faraday's constant	C mol^{-1}	9.65×10^4	55-58, 63, 72-74	-

Ul, unitless, Adj, Adjusted (see main text). * Parameters values were re-calculated from [43, 50] assuming $[K^+]_o$, $[K^+]_i$, $[Na^+]_o$ constant.

References:

17 McClenaghan NH, Barnett CR, Flatt PR (1998) Na^+ cotransport by metabolizable and nonmetabolizable amino acids stimulates a glucose-regulated insulin-secretory response. Biochem Biophys Res Commun 249: 299-303.

39 Fridlyand LE, Jacobson DA, Kuznetsov A, Philipson LH (2009) A Model of Action Potentials and Fast Ca^{2+} Dynamics in Pancreatic β-Cells. Biophysical Journal 96: 3126-3139.

43 Fridlyand LE, Tamarina N, Philipson LH (2003) Modeling of Ca^{2+} flux in pancreatic β-cells: role of the plasma membrane and intracellular stores. American Journal of Physiology - Endocrinology And Metabolism 285: E138-E154.

50 Miwa Y, Imai Y (1999) Simulation of spike-burst generation and Ca^{2+} oscillation in pancreatic beta-cells. Jpn J Physiol 49: 353-364.

61 Luo C, Rudy Y (1994) A dynamic model of the cardiac ventricular action potential. I. Simulations of ionic currents and concentration changes. Circulation Research 74: 1071-1096.
